# Best Practices for Developing Linear Models With Multiple Explanatory Variables

**DOI:** 10.1002/ggn2.202500024

**Published:** 2026-02-13

**Authors:** Baidu Li, Xinhai Li

**Affiliations:** ^1^ Biomedical Engineering School of Graduate Studies University of Toronto Toronto Ontario Canada; ^2^ State Key Laboratory of Conservation of Animal Diversity and Pest Management Institute of Zoology Chinese Academy of Sciences Beijing China; ^3^ University of Chinese Academy of Sciences Beijing China

**Keywords:** interaction term, machine learning, model selection, quadratic term, shrinkage methods

## Abstract

Linear models, including t‐test, ANOVA, regression, ANCOVA, and generalized linear models, are foundational tools in statistical analysis. For large datasets, such as those involving tens of thousands of genes and millions of records, numerous advanced methods have been developed to improve both computational efficiency and reliability. Here, we focus on a more general scenario: a linear model with many explanatory variables (e.g., >10) and a moderate sample size (e.g., thousands of observations). This paper provides the best practices for model selection, emphasizing the importance of including two‐way interaction and quadratic terms, which are frequently overlooked in textbooks and classic literature. When dealing with high‐dimensional data, we recommend using random forest for initial variable screening, followed by subset selection methods such as stepwise regression. Model selection can be guided by criteria like AIC, BIC, adjusted R^2^, and Mallows’ Cp, or by cross‐validation. Shrinkage methods such as the lasso and ridge regression improve model fitting by penalizing coefficient size. Dimension reduction techniques such as Principal Components Regression (PCR) and Partial Least Squares (PLS) provide alternatives for managing high‐dimensional data through uncorrelated component transformation. We provided R code along with detailed descriptions for all analyses, establishing a systematic approach to developing linear models.

## 1 Objectives

Linear models are the most widely used tools in genetics, biology, and the general sciences. However, many researchers fail to apply them correctly. One key reason is that popular textbooks often fail to clarify the essential principles of linear model application, such as including interaction and quadratic terms; another is that traditional approaches do not take advantage of modern machine learning algorithms. In this paper, we present a best‐practice approach to conducting such analyses, accompanied by examples with R code Supporting Information.

## 2 Linear Models

A linear model is a mathematical model that describes the relationship between a dependent variable (often denoted as *Y*) and one or more explanatory variables (often denoted as *X_1_*, *X_2_*, …, *X_p_*) using a linear function [1, 2]. In its simplest form, a linear model can be expressed as:

Y=β0+β1X1+···+βpXp+ε

where *β_0_, β_1_, β_2_, …, β_p_* are model coefficients, *Xs* are explanatory variables, and *ε* represents model errors.

Linear models span from the t‐test to logistic regression, including ANOVA for categorical independent variables and regression for continuous independent variables, forming the main part of basic statistical analysis (Table 1).

**TABLE 1:** Types of linear models.

Models	Dependent variable Y	Independent variables Xs	
		Number of categorical variables	Number of continuous variables
General linear models			
Z test, t test	Continuous variable	1	0
ANOVA	Continuous variable	≥1	0
Linear regression	Continuous variable	0	≥1
ANCOVA	Continuous variable	≥1	≥1
Generalized linear models			
Logistic regression	0/1	≥0	≥0
Poisson regression	Count data (variance = mean)	≥0	≥0
Negative binomial regression	Count data (variance > mean)	≥0	≥0

The fundamental concept of linear models is that the effects of the explanatory variables (*Xs*) on the dependent variable (*Y*) can be added together [3, 4]. This approach provides an approximate estimation of the variance in *Y*, particularly useful when the exact mechanism underlying the relationship between *Xs* and *Y* is not fully understood. Through this additive framework, linear models offer a straightforward and interpretable means to analyze and predict how changes in the independent variables collectively influence the dependent variable.

## 3 New Insights for Model Selection

In genome‐wide association studies (GWAS) and other omics research, investigators routinely analyze tens of thousands of genes, proteins, or metabolites, resulting in large, high‐dimensional datasets. To address the computational and statistical challenges posed by such data, substantial efforts have been devoted to optimizing linear models for greater efficiency and robustness. For instance, Zhang et al. [5] introduced a compressed mixed linear model (MLM) that reduces the effective sample size by clustering observations into groups. In the context of imbalanced data, Cook et al. [6] proposed advanced weighting schemes within a fixed‐effects meta‐analysis framework to improve the performance of linear models with imbalanced data. When dealing with massive‐scale analyses, such as testing billions of transcript–SNP pairs, Shabalin [7] developed Matrix eQTL (Expression Quantitative Trait Loci), which achieves computational efficiency through specialized preprocessing and by reformulating the most intensive computations as large matrix operations. More recently, Hai et al. [8] proposed a two‐step Bayesian linear mixed model framework (TBLMM) for risk prediction using multi‐omics data. Among widely adopted software tools, the R package limma (Linear Models for Microarray Data), originally designed for gene expression microarrays but now extensively used for RNA‐seq, proteomics, methylation arrays, and other high‐dimensional genomic assays, stands out for its ability to fit linear models and apply empirical Bayes moderation of variances, thereby enhancing statistical inference, especially in settings with limited sample sizes [9]. Another relevant tool is the R package lmms, which is specifically tailored for analyzing time‐course omics data [10].

In this study, we focus on a more general scenario: a linear model with a relatively large number of explanatory variables (e.g., >10) and a moderate sample size (e.g., hundreds to thousands of observations). Our goal is to establish best practices for model selection using the most employed techniques.

For an exploratory study, researchers typically gather an extensive array of variables to explain the variance in their variable of interest [11]. This approach, however, often leads to the inclusion of numerous irrelevant variables that should be eliminated during the model selection process. When dealing with a high number of continuous explanatory variables, the issue of multicollinearity becomes inevitable, typically resulting in a complex pattern of X‐Y relationships [12]. Nevertheless, many machine learning algorithms are robust to severe multicollinearity and can effectively identify and exclude unrelated variables [13, 14].

Interaction terms and quadratic terms represent important patterns of linear models; however, they are frequently overlooked in model selection processes. In this paper, we used examples to clarify the effects of interaction terms and quadratic terms.

Linear models have three major approaches for subsequent model selection [3]:

- Subset Selection: This involves selecting a subset of predictors that contribute most significantly to the model.
- Shrinkage Methods: Including ridge regression and lasso regression, these methods help in reducing model complexity by penalizing the magnitude of coefficients.
- Dimension Reduction: Techniques such as Principal Component Regression (PCR) and Partial Least Squares (PLS) regression transform the original predictors into a smaller set of uncorrelated components that still capture most of the variability in the data.

### 3.1 Using Random Forest for Initial Variable Selection

When dealing with a large number of explanatory variables (e.g., over 10), random forest emerges as an excellent option for variable selection [15]. This method leverages tree models capable of efficiently managing thousands of explanatory variables [16]. Random forest is particularly robust in the presence of multicollinearity, and it excels at quantifying interaction effects and nonlinear relationships [17]. Users can identify the most relevant variables by examining their importance indices, making it a powerful tool for filtering unrelated variables.

**FIGURE 1:**
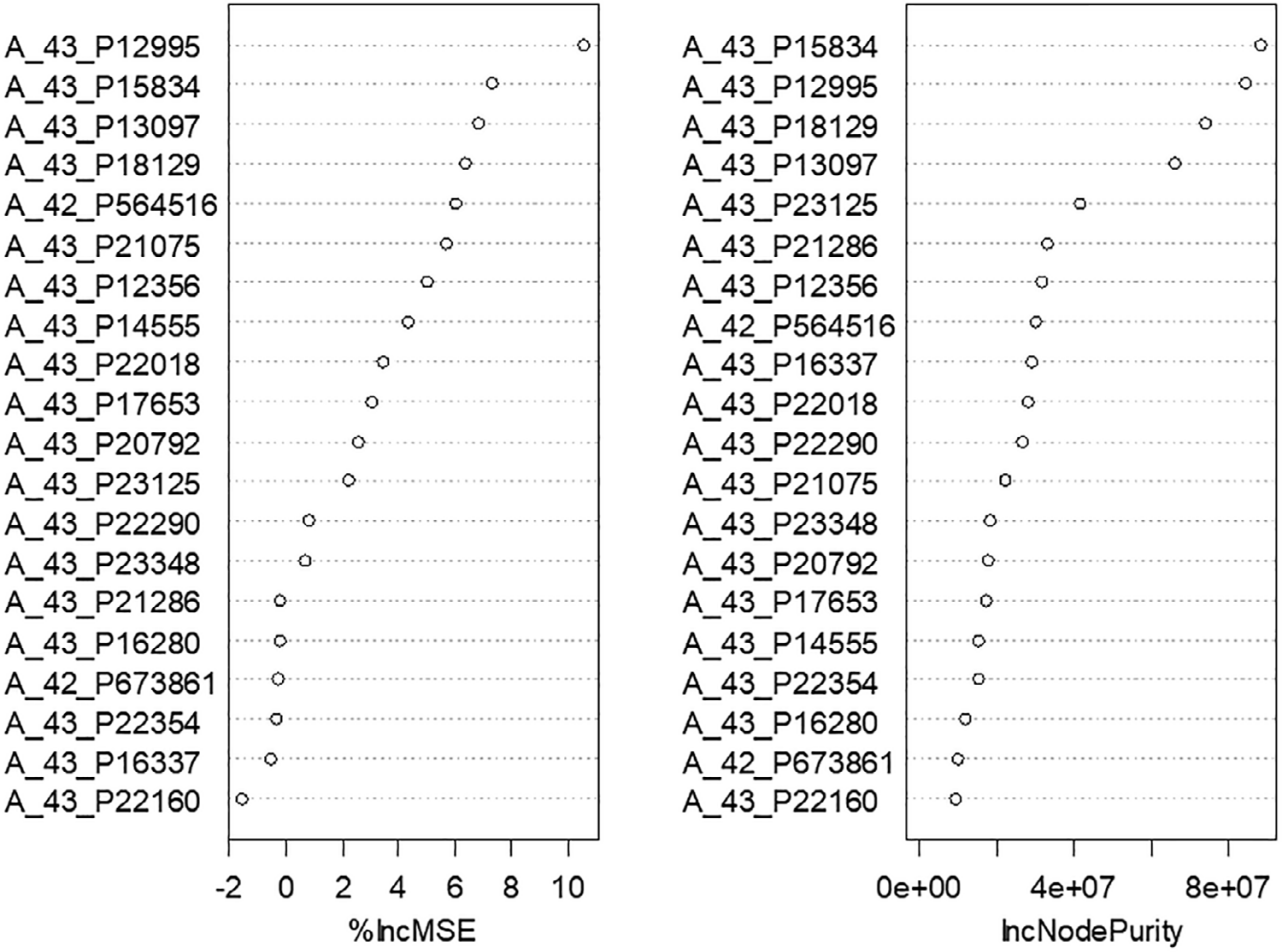
The weight of variables (expression levels of 20 genes) in the liver.toxicity dataset to explain the extent of liver damage. %IncMSE Measures how much a variable's permutation (random shuffling) reduces model accuracy (classification) or increases prediction error (regression). IncNodePurity Measures a variable's contribution to node purity in classification trees (or variance reduction in regression).

**FIGURE 2:**
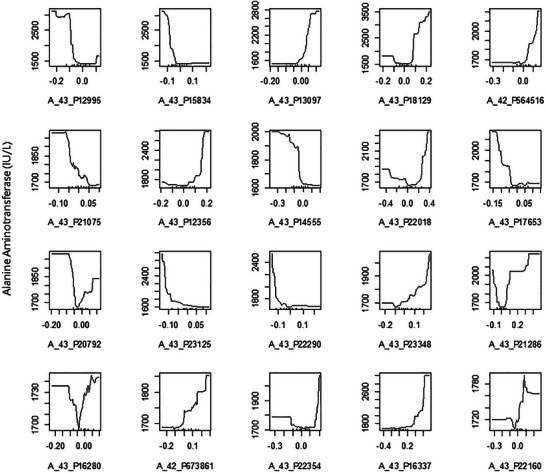
Partial plots illustrating the effect of expression levels of 20 genes on the severity of liver damage. The variables are ordered by importance (based on %IncMSE) from top left to bottom right.

The randomForest function in the randomForest package is one of the most widely used tools for building random forest models [18]. It generally performs well with its default settings, making it a popular choice for both beginners and experienced users in machine learning tasks [19]. The contributions of the variables (Figure 1) and their partial effects (Figure 2) can be visualized using the randomForest package (Algorithms 1, 2, 3, 4, 5, 6, 7, 8, 9, 10, 11).

Algorithm 1 **Using Random Forest to initially select explanatory variables.**

**Table:** 

The R code is:
library(randomForest) # [18]
library(mixOmics) # [20], use this package to access the liver.toxicity dataset
data(liver.toxicity) # load the dataset
D <‐ liver.toxicity$gene[, 1:20] # expression levels of the first 20 genes of 64 rats
D1 <‐ liver.toxicity$clinic # clinical variables of 64 rats
# The variable ALT.IU.L. (Alanine Aminotransferase (IU/L)) is one key clinical variable,
# its high levels indicate hepatocellular injury (liver cell damage)
D = data.frame(ALT.IU.L. = D1$ALT.IU.L., D) # combine a clinical variable and gene expression data
# Using gene expression data (20 genes of 64 rats) to explain the extent of liver damage
RF <‐ randomForest(ALT.IU.L. ∼ A_43_P14555 + A_43_P22290 + A_43_P20792 + A_43_P21286 +
A_43_P12995 + A_43_P15834 + A_43_P12356 + A_42_P564516 + A_43_P22018 +
A_43_P21075 + A_43_P23125 + A_43_P17653 + A_43_P18129 + A_43_P16337 +
A_43_P22160 + A_43_P23348 + A_43_P22354 + A_43_P16280 + A_43_P13097 +
A_42_P673861,
data = D, importance = TRUE, ntree = 1000)
RF # show % Variance explained
varImpPlot(RF, main='') # Figure 1 The weight of the 20 variables (expression levels of 20 genes)
imp <‐ importance(RF) # Derive variable importance indices
impvar <‐ rownames(imp)[order(imp[, 1], decreasing=TRUE)] # sort importance
# Figure 2 Partial plots
op <‐ par(mfrow=c(4, 5),mar=c(4,4,2,2))
for (i in seq_along(impvar)) partialPlot(RF, D, impvar[i], xlab=impvar[i], ylab='', main='')

Based on the percentage increase in mean square error (%IncMSE), genes ranked below A_43_P23125 in Figure 1 (left panel) can be excluded from the analysis due to their weak association with the variable ALT.IU.L. Such a selection may be subjectively determined, as there is no widely recognized rule available.

The genes A_43_P22018, A_43_P20792, and A_43_P16280 exhibit a unimodal effect on liver damage, suggesting that a quadratic term for the expression levels of those genes should be considered in the linear model.

The selected variables might be correlated, so multicollinearity must be checked before developing a linear model. The most effective method for detecting multicollinearity is the variance inflation factor (VIF), which assesses the dependence of a variable on all other variables, whereas correlation analysis only reveals pairwise relationships. The code for calculating the variance inflation factor is:

Algorithm 2 **Using the Variance Inflation Factor (VIF) to assess multicollinearity.**

**Table:** 

X = D[, paste(impvar[1:12], sep=",")] # select 12 most relevant genes
library(car) # the package for calculating variance inflation factor (VIF) [21]
Dat = data.frame(ALT = D$ALT.IU.L., X) # combine Y and Xs
vif(lm(ALT ∼., data=Dat)) # VIF for every explanatory variable

The VIF values for all 12 variables are below 5, indicating that multicollinearity is not a concern and the variables can be considered sufficiently independent. If some variables had VIF values exceeding 5, it would be advisable to remove one or more of them to mitigate multicollinearity [21]. This can be assessed using code such as vif(lm(ALT ∼ A_43_P12995 + A_43_P15834 + A_43_P13097, data = Dat)). When removing variables, it is not necessary to start with the one having the highest VIF; eliminating any correlated variable may reduce the VIF values of others. This allows researchers to strategically retain variables of primary interest while still addressing multicollinearity.

### 3.2 Concept of the Full Model: Importance of the Two‐Way Interaction and Quadratic Terms

Linear models provide a basic estimation of the relationship between explanatory variables and the dependent variable, and therefore should not incorporate complex patterns such as three‐way interactions, cubic terms, or higher‐order terms [2, 22]. However, two‐way interactions and quadratic terms are essential and must be included to capture at least some of the more nuanced nonlinear relationships [22, 23, 24].

A two‐way interaction term in a linear model indicates that the effect of one explanatory variable on the dependent variable depends on the level of another explanatory variable. In other words, the interaction effect reflects a nonlinear relationship, where the combined influence of two variables on the outcome is not simply the sum of their individual effects. Interaction effects are commonly observed in biological research [25]. For example, phenotypic traits in wildlife, such as body size, can result from interactions between taxonomic group and environmental temperature [26], and trait expression is frequently shaped by genotype‐by‐environment interactions [27].

Quadratic terms in linear models represent a unimodal (U‐shaped or anti‐U‐shaped, or a segment thereof) relationship between a predictor variable and the response variable. This nonlinear pattern indicates that the outcome increases (or decreases) with the predictor up to a certain point (the peak or trough), after which the relationship reverses. Such relationships are common in natural systems. For example, enzyme activity often reaches a maximum at an optimal temperature, such as 37 °C, and declines at higher or lower temperatures. Similarly, plant growth rate typically peaks at a neutral soil pH (around pH 7), decreasing under more acidic or alkaline conditions.

Surprisingly, many classic textbooks in statistics and data analysis seldom emphasize the importance of interaction and quadratic terms in linear models (Table 2). For instance, *Multivariate Data Analysis* [28], a highly influential textbook cited 158,653 times (Google Scholar, accessed August 22, 2025), treats quadratic and cubic terms collectively as “curvilinear effects” and defines interaction as a moderating effect. Other popular textbooks such as *Biometry (Fourth Edition)* [29] and *Biostatistical Analysis (Fifth Edition)* [4] also mixed quadratic terms with higher order terms. As a result, researchers often overlook all nonlinear terms in linear models, perceiving them as unnecessarily complex.

**TABLE 2:** Coverage of two‐way interaction and quadratic terms in most influential textbooks.

Books	Quadratic term	Two‐way interaction term	Citations
Multivariate Data Analysis (Eighth Edition)	Mixed with cubic terms	Mixed with three‐way interactions	[28]
Biostatistical Analysis (Fifth Edition)	Mixed with cubic terms	Included	[4]
Biometry (Fourth Edition)	Mixed with cubic terms	Included for ANOVA, ignored for regression	[29]
Modern Applied Statistics with S (Fourth Edition)	Included	mixed with three‐way interactions	[30]
Mathematical Statistics and Data Analysis (Third Edition)^a^	Ignored	/	[31]
Statistical inference (Second Edition)^a^	Ignored	/	[32]
Biostatistical Methods	Ignored	Included	[33]
Mixed Effects Models and Extensions in Ecology with R	Mixed with other nonlinear terms	Mixed with three‐way interactions	[34]
Hierarchical Modeling and Inference in Ecology. The Analysis of Data from Populations, Metapopulations and Communities	Included	Included	[35]
Extending the Linear Model with R	Mixed with cubic terms	Included	[22]
Generalized Linear Mixed Models Modern Concepts, Methods, and Applications	Included	Included	[36]
Statistical methods for biostatistics and related fields	Ignored	Ignored	[37]
The Statistics of Gene Mapping	Ignored	Included	[38]
Theory and Mathematical Methods for Bioinformatics	Ignored	Ignored	[39]
A Modern Introduction to Probability and Statistics: Understanding Why and How^a^ ^)^	Ignored	/	[40]

^a^ These books do not include multiple variate analysis.

Higher‐order terms, such as cubic effects or three‐way interactions, introduce substantial complexity and are often difficult to interpret meaningfully [22]. For example, a three‐way interaction such as “the effect (Y, e.g. blood pressure) of drugs (X1) on gender (X2) depends on age (X3)” is often difficult to interpret and may lack clear practical significance. We suggest that linear models should avoid overly complex nonlinear or interaction terms.

A few books adequately address interaction and quadratic terms in linear models (e.g. [35, 36])(Table 2), yet none explain why such terms are necessary, or why higher‐order interactions and polynomial terms should generally be avoided.

We propose the full model framework for linear models, which included quadratic terms and two‐way interactions, as well as all linear terms. These terms capture essential nonlinear and synergistic relationships in real‐world data without sacrificing model interpretability. A full model should be followed by applying model selection techniques to eliminate terms that contribute little. When there are three continuous explanatory variables, the R code for constructing the full model is as follows:

$$\mathrm{lm}\left(Y∼{\left(X1+X2+X3\right)}^{2}+I\left(X1^{2}\right)+I\left(X2^{2}\right)+I\left(X3^{2}\right)\right)$$

This model includes:

- Three linear terms: X1, X2, and X3;
- Three quadratic terms: X1^2^, X2^2^, and X3^2^, applicable for continuous variables;
- Three two‐way interaction terms: X1:X2, X1:X3, and X2:X3.

#### 3.2.1 Interaction Terms

In linear models, interaction effects are conceptualized differently across methods. In multiple regressions, the interaction term is mathematically defined as the product of two explanatory variables, allowing the effect of one variable to depend on the value of another. In Analysis of Variance (ANOVA), interaction terms quantify the non‐additive combined effect of two or more categorical factors, reflecting how the effect of one factor varies across levels of another. In Analysis of Covariance (ANCOVA), interaction terms test whether the relationship between a continuous covariate and the outcome differs across levels of a categorical variable, effectively assessing heterogeneity in slopes between groups.

It is straightforward to check whether interaction terms in linear models significantly contribute to the dependent variable. In the R programming language, the term variable1:variable2 can be used to represent an interaction term in functions such as *lm* for general linear models and *glm* for generalized linear models, no matter the variable1 and variable2 are continious or categorical varaibles.

**FIGURE 3:**
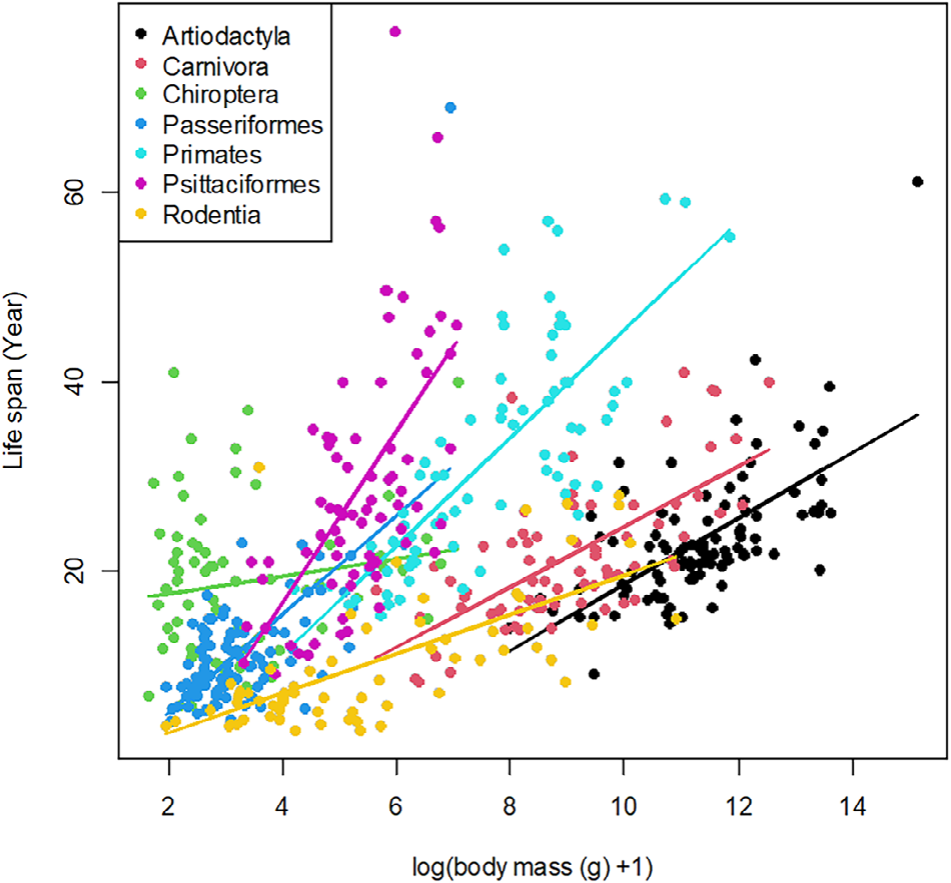
The interaction effect of variables *order* and *mass_g* on *maximum_lifespan_yr*. The relationship between life span and body mass varies across different *orders*.

For the dataset *longevity* in the package Biostatistics [41], the variables *order* (a rank in the biological classification system) and *mass_g* (body mass) exhibit a significant interaction effect on *maximum_lifespan_yr* (Figure 3). Since *order* is a categorical variable with seven levels and *mass_g* is a continuous variable, the model maximum_lifespan_yr ∼ log(D$mass_g+1) * order represents an ANCOVA model. Below is the R code demonstrating how to quantify the interaction effect and visualize the interaction pattern for this ANCOVA model.

Algorithm 3 **Demonstrating the two‐way interaction effect.**

**Table:** 

library(Biostatistics) # [41], to access the dataset longevity
data("longevity")
# select the seven orders with the highest number of species
D = longevity[longevity$order %in% names(sort(table(longevity$order), decreasing=T))[1:7], ]
# Apply a log transformation to body mass to distribute the points more evenly
plot(log(D$mass_g+1), D$maximum_lifespan_yr, col = as.factor(D$order))
# Build a full model with interaction and quadratic terms
model = lm(maximum_lifespan_yr ∼ log(D$mass_g+1) * order + I(log(D$mass_g+1)^2), data = D)
model = step(model) # model selection. The default setting is the backward stepwise selection.
library(car) # for the function Anova()
Anova(model, type="III") # use type III sum of squares to show variance partition
model = update(model,. ∼. ‐I(log(D$mass_g+1)^2)) # remove the quadratic term
pred = predict(model, D[, c('mass_g', 'order')]) # Predict species life span
par(mfrow=c(1,1),mar=c(4.5,4.5,4,2))
# Figure 3 The interaction effect of variables *order* and *mass_g* on *maximum_lifespan_yr*
plot(log(D$mass_g+1), D$maximum_lifespan_yr, col="grey",
xlab="log(body mass (g) +1)", ylab="Life span (Year)")
Dat = cbind(D, pred)
types = names(table(D$order))
for (i in 1:length(types)) {
Data = Dat[Dat$order==types[i], ]
lines(log(Data$mass_g+1), Data$pred, col=i, lwd=2)
points(log(Data$mass_g+1), Data$maximum_lifespan_yr, col=i, pch=16)
}
legend("topleft", types, pch=16, col=c(1:7))

#### 3.2.2 Quadratic Terms

**FIGURE 4:**
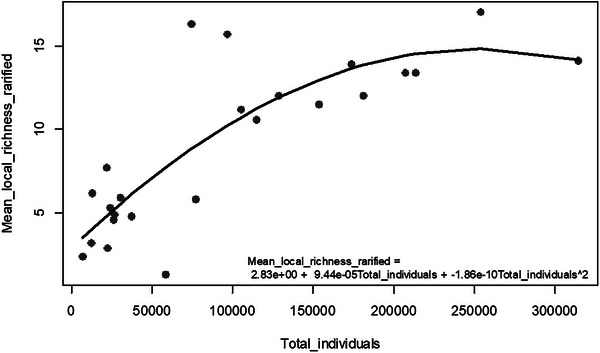
The relationship between *Total_individuals* and *Mean_local_richness_rarified* in the latitude_diversity dataset. The grey points represent the original values of the variables. The black curve illustrates the predicted value of *Mean_local_richness_rarified* when the variable *Total_individuals* is modeled using a linear and a quadratic term.

A quadratic term in linear models indicates the X‐Y relationship forms a parabolic curve. We used a dataset on tree diversity from 24 forest plots in locations ranging from the tropics to northern Europe and the USA [41] as an example. A linear model is developed to explain a biodiversity index, *Mean_local_richness_rarified*, by two variables, *Plot_size_Ha* and *Total_individuals*, and the quadratic term of *Total_individuals* appears to have a significant contribution to the dependent variable (Figure 4).

Algorithm 4 **Demonstrating the effect of a quadratic term.**

**Table:** 

library(Biostatistics)
data("latitude_diversity") # the tree diversity dataset
D = latitude_diversity[, 5:12] # select variables for analysis
# The full model with interaction and quadratic terms
fit = lm(Mean_local_richness_rarified ∼ (Plot_size_Ha + Total_individuals)^2 + I(Plot_size_Ha^2) + I(Total_individuals^2), data = D)
fit = step(fit) # backward stepwise model selection
summary(fit) # show model results, the variable Plot_size_Ha was removed
# Predicted biodiversity
pred = predict(fit, D[, c("Mean_local_richness_rarified", "Total_individuals")])
# Figure 4 The association between the Total_individuals and Mean_local_richness_rarified
with(D, plot(Total_individuals, Mean_local_richness_rarified, pch=16, col="grey40", cex=1.2))
D = cbind(D, pred)
D = D[order(D$Total_individuals), ] # sort observations to plot a smooth curve
lines(D$Total_individuals, D$pred, col=1, lwd=3)
coeff = format(coefficients(fit), digits = 3, scientific = TRUE) # model coefficients
legend("bottomright", paste("Mean_local_richness_rarified = ∖n", coeff[1], " + ", coeff[2],
"Total_individuals", " + ", coeff[3], "Total_individuals^2", sep=""), bty = "n", cex=0.8,
inset = c(0, 0.03))

**FIGURE 5:**
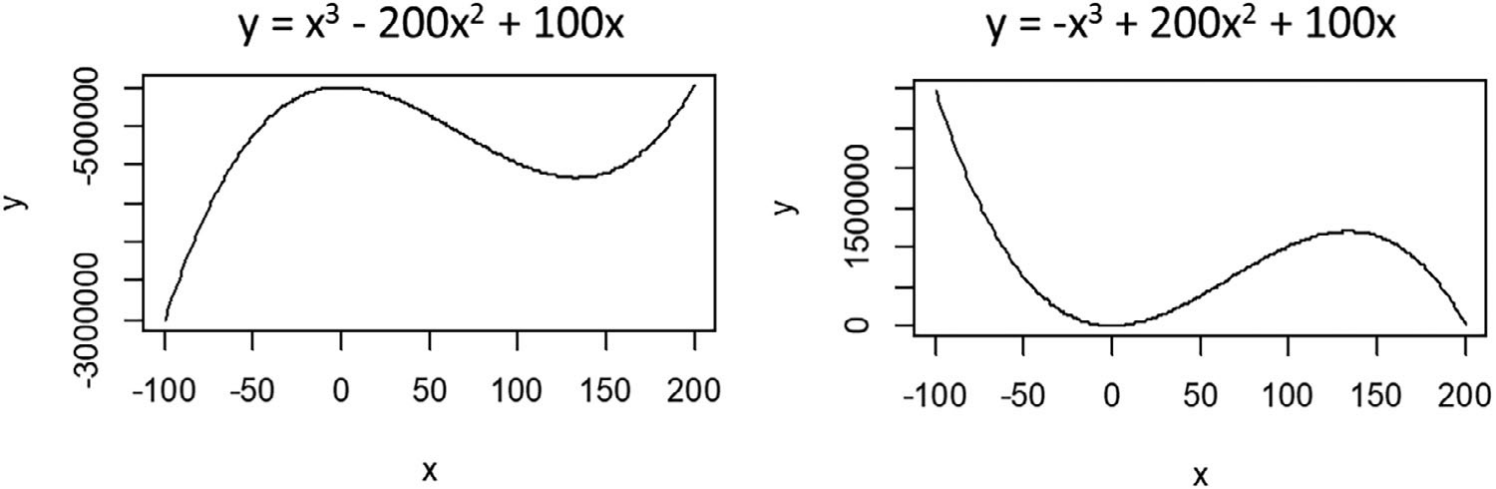
Examples of linear models with cubic terms to illustrate nonlinear X–Y relationships.

A cubic term in a linear model captures a nonmonotonic, S‐shaped relationship between the predictor X and the response Y (Figure 5). Unlike a linear term, which indicates a consistent positive or negative trend; or a quadratic term, which implies a single turning point and an extremum (maximum or minimum); a cubic relationship lacks a simple, interpretable pattern. When multiple predictors include cubic terms, the resulting model becomes highly complex and difficult to interpret. For modeling such higher‐order, nonlinear relationships, generalized additive models (GAMs) are generally more appropriate than traditional linear models [42].

## 4 Basic and Advanced Model Selection

The process of model selection aims to eliminate irrelevant variables and terms in order to determine the optimal combination for fitting the dependent variable. Basic model selection methods include various subset selection approaches, while advanced techniques encompass shrinkage methods such as ridge regression and the lasso, and dimension reduction models such as Principal Components Regression (PCR) and Partial Least Squares (PLS) [3].

### 4.1 Subset Selection

This approach involves selecting a subset of explanatory variables (including interaction and higher‐order terms) that are believed to be related to the dependent variable [43]. Least squares estimation is typically used to reduce the number of variables and terms. It consists of an exhaustive comparison of all potential models as well as a more efficient stepwise model selection process.

#### 4.1.1 Comparison with All Potential Models

In traditional best subset selection, the standard approach involves comparing models that include every possible combination of explanatory variables, along with their two‐way interaction terms and quadratic terms (where applicable). Note that categorical explanatory variables do not have quadratic terms.

For example, with four continuous explanatory variables, the full model would consist of four linear terms, four quadratic terms, and six two‐way interaction terms. This results in a total of 14 terms to evaluate. The number of candidate models is calculated as the sum of combinations of these 14 terms, ranging from selecting 0 terms (the null model) to all 14 terms. Mathematically, this is expressed as $\sum_{0}^{k}\left(\begin{array}{c} p\\ k \end{array}\right)$ = 2^14^ = 16384, where *p* is 14 for this case, and *k* is possible number of terms in the final model, ranging from 0 to *p*. Each potential model needs to be evaluated using criteria such as adjusted R‐squared, AIC, BIC, or cross‐validation error to identify the model that best balances performance and complexity. For computational reasons, best subset selection cannot be applied when the number of explanatory variables is large (e.g. > 4).

#### 4.1.2 Forward Stepwise Selection

Forward stepwise selection serves as a computationally efficient alternative to best subset selection. Unlike the best subset selection method, which evaluates all 2^p^ possible models comprising subsets of the p terms, forward stepwise selection examines a significantly smaller set of models. This process starts with a model that contains no predictors and incrementally adds one predictor at a time until all predictors are included. Specifically, at each step, the predictor that provides the greatest improvement in model fit is added. This approach efficiently narrows down the model choices without the need to evaluate every possible combination.

Forward stepwise selection offers a clear computational advantage over best subset selection. However, while it generally performs well in practical scenarios, there is no guarantee that it will identify the optimal model among all 2^p^ possible models containing subsets of the *p* terms. For example, consider a dataset with three terms *X1*, *X2*, and *X3*. The best one‐variable model includes *X1*, but the best two‐variable model consists of *X2* and *X3*. In this case, forward stepwise selection would fail to select the best possible two‐variable model because it initially selects *X1* for the first model. Consequently, the second model must include *X1* along with one additional variable, thus missing the optimal two‐variable model.

#### 4.1.3 Backward Stepwise Selection

Similar to forward stepwise selection, backward stepwise selection offers an efficient alternative to best subset selection. However, instead of starting with no predictors, it begins with the full least squares model that includes all *p* terms. It then iteratively eliminates the least useful term, one at a time, until an optimal subset of predictors is reached.

#### 4.1.4 Hybrid Approaches

The best subset, forward stepwise, and backward stepwise selection methods typically yield similar yet distinct models when the number of explanatory variables is large. Another alternative involves hybrid approaches that combine elements of both forward and backward stepwise selection. In these hybrid versions, variables are added to the model sequentially, akin to forward selection. However, after each new variable is added, the method may also remove any variables that no longer enhance the model fit. This strategy aims to more closely approximate the results of best subset selection while maintaining the computational efficiency associated with forward and backward stepwise selection processes.

The step() function in the stats package performs stepwise model selection based on the Akaike Information Criterion (AIC). It supports forward, backward, or hybrid (both directions) model selection. When the scope argument is missing, it performs backward stepwise selection. If argument *direction = “both”* is used, step() performs both forward and backward stepwise model selection. The following example uses the expression level of three most relevant genes from the *liver.toxicity* dataset to explain liver damage.

Algorithm 5 **Stepwise model selection.**

**Table:** 

# In Section 4, random forest was used to selection most important genes in dataset liver.toxicity
# The importance of genes has been calculated and stored in impvar
# Select the index for liver damage and the expression level of three most relevant genes data(liver.toxicity) # load the dataset D <‐ liver.toxicity$gene[, 1:20] # expression levels of the first 20 genes of 64 rats D1 <‐ liver.toxicity$clinic # clinical variables of 64 rats D = data.frame(ALT.IU.L. = D1$ALT.IU.L., D)
D = D[, c("ALT.IU.L.", impvar[1], impvar[2],impvar[3])]
names(D) = c("Damage", "G1", "G2", "G3") # rename the variable for simplicity
# The full model
model1 = lm(Damage ∼ (G1 + G2 + G3)^2 + I(G1^2) + I(G2^2) + I(G3^2), data = D)
# backward stepwise model selection
model2 = step(model1)
# both directions
model3 = step(model1, direction = "both")
# compare the three models
anova(model1, model2, model3)

#### 4.1.5 Model Performance Indices: Cp, AIC, BIC, and Adjusted R^2^

A model containing all possible terms will always achieve the smallest residual sum of squares (RSS) and the largest R^2^ value, as these metrics reflect performance on the training data (the observed data) [3, 43]. However, these quantities measure training error, which often underestimates test error (a model's true performance on unseen data). Consequently, RSS and R^2^ are unsuitable for selecting the best model when comparing candidates with varying numbers of terms. To address this limitation, several techniques adjust training error metrics to penalize excessive model size. These methods enable fair comparisons among models with different numbers of terms. Four widely used approaches include:

- C_p_ (Mallows’ Statistic)
- Akaike Information Criterion (AIC)
- Bayesian Information Criterion (BIC)
- Adjusted R^2^

These criteria balance model fit and complexity, favoring models that generalize well to new data rather than merely optimizing for training performance.

For a linear model containing *d* terms, the C_p_ estimate of test mean squared error (MSE) using least square method is computed using the equation

$$C_{p}=\frac{RSS+2d{\hat{\sigma }}^{2}}{n}$$

where RSS is the residual sum of squares, *n* is the number of observations, ${\hat{\sigma }}^{2}$ is an estimate of the variance of the error *Ɛ* associated with each observation. While making comparisons, number *d* is the difference of number of terms in two models [44].

Typically, ${\hat{\sigma }}^{2}$ is estimated using the full model that contains all terms. The *C_p_* statistic adds a penalty of $2d{\hat{\sigma }}^{2}$ to the training RSS to account for the fact that the training error tends to underestimate the test error. This penalty increases with the number of terms in the model, which adjusts for the corresponding decrease in training RSS. As a result, *C_p_* provides an unbiased estimate of the test MSE. Consequently, the *C_p_* statistic tends to be smaller for models with lower test errors. Therefore, when selecting the best model from a set, the one with the lowest *C_p_* value should be chosen.

The AIC is a measure used for model selection among a set of candidate models. It provides a means to compare different models fitted to the same dataset by balancing goodness of fit and model complexity. For a given model, AIC is calculated as:

$$AIC=2k\;-\;2\ln(\hat{L})$$

where *k* is the number of parameters in the model, and $\hat{L}\;$ is the maximum value of the likelihood function for the estimated model.

In the context of linear regression models fitted with least squares, AIC can be expressed alternatively as:

AICleast_square=2k+ln(RSS/n)n

where *n* is the number of observations, k is the number of parameters in the model, and *RSS* is the residual sum of squares [45].

The Bayesian Information Criterion (BIC) is another criterion used for model selection among a set of candidate models. Like the AIC, BIC balances model fit and complexity but with a stronger penalty for models with more parameters. This makes BIC particularly useful when you want to avoid overfitting by favoring simpler models, especially in cases where the sample size is large. For a given model, BIC is calculated as:

$$BIC=k\;\ln\left(n\right)\;-\;2\ln\left(\hat{L}\right)$$

where *n* is the number of observations, *k* is the number of parameters in the model, and $\hat{L}\;$ is the maximum value of the likelihood function for the estimated model [46].

For linear regression models fitted with least squares, BIC can be expressed as:

BICleast_square=kln(n)+nln(RSS/n)

where *n* is the number of observations, *k* is the number of parameters in the model, and *RSS* is the residual sum of squares.

Adjusted R^2^ is a modified version of R^2^ that adjusts for the number of terms in a linear model. While R‐squared always increases with the addition of more terms, whether they improve the model's fit or not, adjusted R^2^ compensates for the number of terms in the model. It incorporates a penalty for adding terms that do not contribute significantly to the model. This makes it a better tool for comparing models with different numbers of terms.

The formula for adjusted R^2^ is:

$$AdjustedR^{2}=1-\frac{RSS/\left(n-k-1\right)}{TSS/\left(n-1\right)}$$

where *n* is the number of observations, *k* is the number of parameters in the model, and *RSS* is the residual sum of squares, *TSS* is the total sum of squares of the dependent variable [29].

Unlike C_p_, AIC, and BIC, where a smaller value indicates a model with low test error, a large value of adjusted Adjusted R^2^ indicates a model with a small test error. Adjusted R^2^ is more commonly used to quantify model performance, whereas AIC and BIC are regularly used for model selection.

#### 4.1.6 Cross‐Validation

The test error of linear models can be directly compared using cross‐validation [47]. By computing cross‐validation error for each model under consideration, researchers can select the model with the smallest estimated test error. This approach has an advantage over AIC, BIC, C_p_, and Adjusted R^2^ because it provides a direct estimate of the test error and makes fewer assumptions about the true underlying model [48]. Additionally, this method is applicable to a broader range of model selection tasks, including scenarios where determining the model's degrees of freedom (such as the number of predictors) or estimating the error variance is challenging.

In the past, performing cross‐validation was computationally prohibitive for problems involving large numbers of predictors (large p) and/or large sample sizes (large n). Consequently, methods like AIC, BIC, Cp, and Adjusted R^2^ were more attractive for model selection. However, with the advent of fast computers, the computational demands of cross‐validation are now rarely a concern. As a result, cross‐validation has become a highly attractive approach for model selection [49].

The following code performs 5‐fold cross‐validation to compare four models using the *mtcars* dataset embodied in the *dataset* package in R.

Algorithm 6 **Model comparisons based on cross‐validation.**

**Table:** 

set.seed(123) # Set seed for reproducibility
data = mtcars
k <‐ 5 # Define k (number of folds)
folds <‐ sample(1:k, nrow(data), replace = TRUE) # Create folds
# Define candidate models (update formulas as needed)
models <‐ list(
mpg ∼ wt,
mpg ∼ wt + hp,
mpg ∼ wt + hp + disp,
mpg ∼ wt * hp
)
# Matrix to store cross‐validation errors
cv_errors <‐ matrix(NA, nrow = k, ncol = length(models))
# Perform k‐fold cross validation
for (i in 1:k) {
train_data <‐ data[folds != i, ]
test_data <‐ data[folds == i, ]
for (j in 1:length(models)) {
# Fit model
fit <‐ lm(formula = models[[j]], data = train_data)
# Predict and compute MSE
pred <‐ predict(fit, newdata = test_data)
cv_errors[i, j] <‐ mean((test_data$mpg ‐ pred)^2) # MSE
}
}
# Average errors across folds
avg_mse <‐ colMeans(cv_errors)
# Select the best model
best_model_idx <‐ which.min(avg_mse) # the fourth model
best_model <‐ models[best_model_idx] # mpg ∼ wt * hp
print(paste("Best model:", format(best_model)))

#### 4.1.7 Popular Packages for Model Selection and Variance Partition

Several packages have been developed and are commonly used for model selection in R. In this paper, we recommend the *MuMIn* and *dominanceanalysis* packages due to their clarity and popularity.

The *MuMIn* package in R facilitates multi‐model inference by providing functions for model selection, averaging, and comparison. Starting from a global model, it can automatically generate all possible submodels using the dredge function, rank them based on information criteria such as AICc. To account for model uncertainty, it computes model‐averaged parameter estimates via *model.avg*. Importantly, MuMIn supports a wide range of model classes, including linear (*lm*), generalized linear (*glm*), additive (*gam*) and mixed‐effects models (*lmer* and *glmer*), making it a versatile choice for robust statistical inference across diverse modeling frameworks [50].

If a model includes three continuous explanatory variables (only continuous variables have quadratic terms), and all two‐way interactions and quadratic terms are included, the full model contains nine terms in total, the *dredge* function evaluates 2^9^ = 512 potential models. When the number of continuous explanatory variables increases to four, and all two‐way interactions and quadratic terms are included, the number of candidate models rises to 2^14^ = 16,384. The *model.avg* function can then integrate over a set of top‐performing models, accounting for model uncertainty and providing a more robust prediction of the dependent variable [50].

The following code use the liver damage data, *liver.toxicity*, to perform a multiple regression and compare all potential models. The model coefficients of the top six models are listed in Table 3.

**TABLE 3:** The coefficients and model fit indices of the top six models using the function *dredge*.

ID	(Int)	G1	G1^2^	G2	G2^2^	G3	G3^2^	G1:G2	G1:G3	G2:G3	df	logLik	AICc	Delta	Weight
246	735.7	−16510		−2675		23680	166400	189600	−232300		8	−593.708	1206	0	0.408
502	721.4	−14550		−2953		22700	172100	215100	−196400	−96110	9	−593.358	1208	2.02	0.149
214	1215	−12730		−3676		16770		200700	−136200		7	−596.285	1208.6	2.54	0.115
248	762.7	−18620	−25220	−1394		23770	167200	215100	−231700		9	−593.629	1208.6	2.56	0.114
86	1121	−10680		−6249		20650		186400			6	−597.612	1208.7	2.66	0.108
254	712.6	−16330		−3091	7268	23670	166400	185600	−231500		9	−593.698	1208.7	2.7	0.106

Algorithm 7 **Application of the MuMIn package for model comparison.**

**Table:** 

library(MuMIn)
library(mixOmics); data(liver.toxicity)
# Select three relevant genes based on their importance calculated in Section 4
D <‐ liver.toxicity$gene[, c("A_43_P12995", "A_43_P15834", "A_43_P13097")]
Damage <‐ liver.toxicity$clinic$ALT.IU.L. # the dependent variable
D <‐ cbind(Damage, D); names(D)[2:4] = c("G1","G2","G3")
# The full model
M1<‐ lm(Damage ∼ (G1 + G2 + G3)^2 + I(G1^2) + I(G2^2) + I(G3^2), data = D, na.action = na.fail)
M2<‐ step(M1) # model selection
model.sel(M1, M2) ## ranked with AICc by default
fit.all <‐ dredge(M1) # comparison among all potential models
fit.all[1:6, ] # Table 3. the top 6 models
# Models with delta.aicc < 4 (delta.aicc of the best model is 0)
model.best <‐ get.models(fit.all, subset = delta < 4)
model.avg <‐ model.avg(model.best)
summary(model.avg)
# Predict values of the dependent variable using the average model (with averaged coefficients)
pred <‐ predict(model.avg, newdata=D, full = FALSE)

The *dominanceanalysis* package in R implements dominance analysis to assess the relative importance of predictors in regression models by decomposing model fit (e.g., R^2^) across all possible predictor subsets, providing measures of general, conditional, and complete dominance that help rank variables based on their contribution to explained variance, making it especially useful for interpreting complex models with correlated predictors [51].

For simplicity, we use the expression levels of two genes to explain liver damage through a bivariate regression, and the contribution of each term of predictors in the context of every possible combinations of terms are shown in Table 4. The *dominanceanalysis* package can also be applied to generalized linear models [52] and hierarchical linear models [53].

**TABLE 4:** Dominance analysis quantifies the contribution (in terms of explained R^2^) of each term of predictors in the bivariate regression in the context of every possible combinations of variables and model terms (the column *model* in the table).

Model	Level	fit	G1	G2	G1^2^	G2^2^	G1:G2
1	0	0	0.087	0.086	0.108	0.022	0.217
G1	1	0.087		0.029	0.031	0.012	0.144
G2	1	0.086	0.03		0.073	0.044	0.138
I(G1^2)	1	0.108	0.01	0.05		0.009	0.11
I(G2^2)	1	0.022	0.077	0.107	0.095		0.195
G1:G2	1	0.217	0.014	0.007	0.001	0.001	
Average level 1	1		0.033	0.048	0.05	0.016	0.147
G1+G2	2	0.116			0.042	0.029	0.117
G1+I(G1^2)	2	0.118		0.04		0.009	0.115
G1+I(G2^2)	2	0.099		0.045	0.028		0.132
G1+G1:G2	2	0.231		0.002	0.002	0	
G2+I(G1^2)	2	0.158	0			0.024	0.067
G2+I(G2^2)	2	0.129	0.015		0.053		0.098
G2+G1:G2	2	0.224	0.009		0.002	0.003	
I(G1^2)+I(G2^2)	2	0.118	0.009	0.065			0.101
I(G1^2)+G1:G2	2	0.218	0.015	0.007		0.001	
I(G2^2)+G1:G2	2	0.218	0.014	0.009	0.001		
Average level 2	2		0.01	0.028	0.021	0.011	0.105
G1+G2+I(G1^2)	3	0.158				0.025	0.075
G1+G2+I(G2^2)	3	0.144			0.039		0.09
G1+G2+G1:G2	3	0.232			0.001	0.002	
G1+I(G1^2)+I(G2^2)	3	0.127		0.056			0.106
G1+I(G1^2)+G1:G2	3	0.233		0		0	
G1+I(G2^2)+G1:G2	3	0.231		0.003	0.002		
G2+I(G1^2)+I(G2^2)	3	0.182	0.001				0.047
G2+I(G1^2)+G1:G2	3	0.225	0.008			0.004	
G2+I(G2^2)+G1:G2	3	0.227	0.007		0.002		
I(G1^2)+I(G2^2)+G1:G2	3	0.219	0.015	0.011			
Average level 3	3		0.008	0.017	0.011	0.008	0.08
G1+G2+I(G1^2)+I(G2^2)	4	0.183					0.051
G1+G2+I(G1^2)+G1:G2	4	0.233				0.001	
G1+G2+I(G2^2)+G1:G2	4	0.234			0		
G1+I(G1^2)+I(G2^2)+G1:G2	4	0.233		0.001			
G2+I(G1^2)+I(G2^2)+G1:G2	4	0.229	0.005				
Average level 4	4		0.005	0.001	0	0.001	0.051
G1+G2+I(G1^2)+I(G2^2)+G1:G2	5	0.235

Algorithm 8 **Application of the dominanceanalysis package for quantifying effects.**

**Table:** 

library(dominanceanalysis)
fit = lm(Damage ∼ (G1 + G2)^2^ + I(G1^2^) + I(G2^2^), data=D)
effects <‐ dominanceAnalysis(fit)
summary(effects) # Table 3

### 4.2 Shrinkage

Shrinkage methods fit a model that includes all terms, but the estimated coefficients are adjusted closer to zero compared to those obtained through least squares estimation [54]. This approach mimics the effect of model selection and helps in reducing the model's variance. Depending on the specific shrinkage technique applied, some coefficients may be estimated as exactly zero. Two prominent techniques for shrinking regression coefficients toward zero are Ridge Regression and the lasso. These methods not only mitigate the risk of overfitting by penalizing large coefficients but also improve the model's predictive accuracy on new data.

#### 4.2.1 Ridge Regression

Ridge regression is closely related to least squares regression, but with an important difference: the coefficients are estimated by minimizing a modified objective function, which is the sum of the residual sum of squares (RSS) and a shrinkage penalty term:

$$\sum_{i=1}^{n}\left(y_{i}-\hat{y_{i}}\right)+\lambda \sum_{j=1}^{p}\beta_{j}^{2}$$

where λ >= 0 is a tuning parameter, to be determined separately [55].

This equation balances two competing objectives. Like least squares regression, ridge regression aims to find coefficient estimates that provide a good fit to the data by minimizing the residual sum of squares (RSS). However, it includes a second term, the shrinkage penalty, which becomes smaller when the coefficients *β_1_*, *β_2_*, … *β_p_* are close to zero. This penalty encourages the coefficient estimates to shrink toward zero, effectively reducing model complexity.

The tuning parameter λ controls the trade‐off between these two objectives: fitting the data well and keeping the coefficients small. When λ = 0, the penalty has no effect, and ridge regression yields the same estimates as ordinary least squares. As λ increases, the influence of the penalty grows, causing the coefficient estimates to move closer to zero.

Choosing an appropriate value of λ is crucial for achieving optimal performance, and techniques like cross‐validation are commonly used to select the best value. The cv.glmnet() function in the glmnet package selects the optimal λ through k‐fold cross‐validation: it fits the model across a grid of λ values, computes the average prediction error (typically mean squared error) for each λ by training on k−1 folds and validating on the held‐out fold, then chooses either lambda.min, the λ with the lowest cross‐validated error, or lambda.1se, the largest λ within one standard error of the minimum, favoring a more regularized (less overfit) model.

The shrinkage penalty does not applied to the intercept *β_0_*. We aim to shrink the estimated relationship between each terms and the dependent variable; however, we do not apply shrinkage to the intercept, as it simply reflects the mean value of the response when all terms are zero.

Ridge regression's advantage over least squares regression lies in its ability to navigate the bias‐variance trade‐off [56]. As the tuning parameter λ increases, the flexibility of the ridge regression model decreases. This reduction in flexibility leads to lower variance but introduces some bias into the coefficient estimates. At λ = 0, which corresponds to the least squares estimates, there is no bias but potentially high variance. As λ increases, the shrinkage of the ridge regression coefficients reduces the variance of the predictions, albeit at the cost of a slight increase in bias. This trade‐off often results in improved model performance and more stable predictions.

In general, when the relationship between the dependent variable and the terms is approximately linear, least squares estimates tend to have low bias but can exhibit high variance. This means that small changes in the training data can lead to large fluctuations in the estimated coefficients. The problem becomes especially pronounced when the number of terms *p* is close to the number of observations n, in which case the least squares estimates become highly unstable. When p > n, the least squares solution is not uniquely defined, making it impossible to compute without additional constraints. In contrast, ridge regression remains effective in such scenarios by introducing a small amount of bias in exchange for a substantial reduction in variance. As a result, ridge regression is particularly well‐suited for situations where ordinary least squares would yield highly variable estimates [56].

Ridge regression also offers significant computational advantages over best subset selection, which requires evaluating up to 2^p^ possible models [55]. As previously discussed, this exhaustive search becomes computationally impractical even for moderate values of p. In contrast, for any given value of λ, ridge regression fits just one model, and the fitting process can be executed relatively quickly. This efficiency makes ridge regression a more scalable solution for datasets with a large number of terms.

The R code for ridge regression of model Murder ∼ (Assault + Rape + UrbanPop)^2 + I(Assault^2) + I(Rape^2) + I(UrbanPop^2) using the US crime data *USArrests* in the base *datasets* package in R. The function cv.glmnet in package glmnet can perform both ridge regression and the lasso [57].

Algorithm 9 **Ridge regression and the lasso.**

**Table:** 

library(glmnet) # for ridge regression and the lasso
data(USArrests) # Violent crime rates by US state
data <‐ USArrests
# Set seed for reproducibility
set.seed(123)
# Split data into training (70%) and testing (30%) sets
train_indices <‐ sample(1:nrow(data), size = 0.7 * nrow(data))
train <‐ data[train_indices, ]
test <‐ data[‐train_indices, ]
# Create model matrices (excluding intercept)
x_train <‐ model.matrix(Murder ∼ (Assault + Rape + UrbanPop)^2 + I(Assault^2) +
I(Rape^2) + I(UrbanPop^2), data = train)[, ‐1]
y_train <‐ train$Murder
x_test <‐ model.matrix(Murder ∼ (Assault + Rape + UrbanPop)^2 + I(Assault^2) +
I(Rape^2) + I(UrbanPop^2), data = test)[, ‐1]
y_test <‐ test$Murder
# Perform ridge regression with cross‐validation
cv_ridge <‐ cv.glmnet(x_train, y_train, alpha = 0) # alpha=0 for ridge
# Plot cross‐validation error versus log(lambda)
plot(cv_ridge)
# Get optimal lambda value
best_lambda <‐ cv_ridge$lambda.min
cat("Optimal lambda:", best_lambda, "∖n")
# Make predictions using best lambda
ridge_pred <‐ predict(cv_ridge, newx = x_test, s = "lambda.min")
# Calculate performance metrics
ridge_rmse <‐ sqrt(mean((ridge_pred—y_test)^2))
ss_res <‐ sum((ridge_pred—y_test)^2)
ss_tot <‐ sum(y_test—mean(y_test)^2)
ridge_rsq <‐ 1 ‐ (ss_res/ss_tot)
# Linear regression
lm_model <‐ lm(Murder ∼ (Assault + Rape + UrbanPop)^2 + I(Assault^2) +
I(Rape^2) + I(UrbanPop^2), data = train)
lm_pred <‐ predict(lm_model, newdata = test)
lm_rmse <‐ sqrt(mean((lm_pred—y_test)^2))
ss_res_lm <‐ sum((lm_pred—y_test)^2)
lm_rsq <‐ 1 ‐ (ss_res_lm/ss_tot)
# Display ridge regression coefficients
cat("∖nRidge Regression Coefficients:∖n")
print(coef(cv_ridge, s = "lambda.min"))
# Compare with linear regression

**Table:** 

cat("∖nRidge Regression Test RMSE:", ridge_rmse)
cat("∖nLinear Regression Test RMSE:", lm_rmse)
cat("∖nRidge Regression Test R‐squared:", ridge_rsq, "∖n")
cat("∖nLinear Regression Test R‐squared:", lm_rsq, "∖n")

Here, the R^2^ value increased from 0.27 for linear regression to 0.44 for ridge regression.

#### 4.2.2 The Lasso

Ridge regression has one notable drawback: unlike best subset, forward stepwise, or backward stepwise selection, which typically result in models that include only a subset of the predictors, ridge regression retains all p terms in the final model. The penalty $\;\lambda \sum_{j=1}^{p}\beta_{j}^{2}$ shrinks all coefficients toward zero, but it does not set any of them exactly to zero (unless λ = ∞). While this may not significantly impact predictive performance, it can complicate model interpretation, especially when the number of terms *p* is large.

The lasso is a more recent alternative to ridge regression that addresses this drawback. The lasso coefficients are obtained by minimizing a similar objective function as in ridge regression, but with a different type of penalty, one that can result in some coefficients being exactly zero [58]. The lasso coefficients minimize the function:

$$\sum_{i=1}^{n}\left(y_{i}-\hat{y_{i}}\right)+\lambda \sum_{j=1}^{p}\left|\beta_{j}\right|$$

The lasso uses $|\beta_{j}|$ to replace the $\beta_{j}^{2}$ in the penalty term. This penalty term encourages sparsity by forcing some coefficient estimates to be exactly zero when the tuning parameter λ is sufficiently large [58]. In this way, the lasso performs variable selection, similar to best subset selection. Consequently, models produced by the lasso are typically more interpretable than those from ridge regression. Researchers refer to these as sparse models, i.e. models that include only a subset of the available terms [59]. Just as in ridge regression, choosing an appropriate value for λ is essential to achieving good performance with the lasso.

Just as subset selection methods require a strategy to determine the best model among those considered, implementing ridge regression and lasso necessitates selecting an optimal value for the tuning parameter λ. Cross‐validation offers a straightforward approach to this task. We start by choosing a grid of λ values, then compute the cross‐validation error for each λ. The value of λ that results in the lowest cross‐validation error is selected as the optimal tuning parameter. Finally, the model is re‐fitted using all available observations and this chosen value of λ.

We used the same data for the lasso, in order to complete model selection base on cross validation errors. The model is: Murder ∼ (Assault + Rape + UrbanPop)^2 + I(Assault^2) + I(Rape^2) + I(UrbanPop^2). The code for the lasso is:

cv_lasso <‐ cv.glmnet(x_train, y_train, alpha = 1) # alpha = 1 for lasso; alpha = 0 for ridge

Compared to ridge regression using the same data, we found that the optimal λ value for ridge regression was 0.35, while for the lasso it was 0.22. The R^2^ values were as follows: 0.27 for ordinary least squares regression, 0.44 for ridge regression, and 0.32 for the lasso.

### 4.3 Dimension Reduction

Dimension reduction involves projecting the original *p* variables into an M‐dimensional subspace, where *M<p*, by computing *M* distinct linear combinations or projections of the variables. These derived features are then used as predictors in a least squares regression model [60].

We now explore a class of techniques that first transform the original predictors and then fit a linear regression model using the transformed variables via ordinary least squares. These methods are commonly referred to as dimension reduction techniques. Among them, Principal Components Regression (PCR) and Partial Least Squares (PLS) are two of the most widely used approaches [61].

Dimension reduction transforms *p* variables under the assumption that they are linearly correlated, so that their linear combination can capture more information than any single variable alone [60]. In this section, we do not advocate for the inclusion of interaction or quadratic terms in the dimension reduction analysis, because introducing non‐linear relationships into the data fundamentally disrupts the assumptions underlying dimension reduction algorithms such as PCA.

#### 4.3.1 Principal Components Regression (PCR)

Principal Components Analysis (PCA) is a widely used technique for deriving a smaller set of uncorrelated features from a large collection of variables [62]. It reduces the dimensionality of an n×p data matrix *X* by identifying directions, called principal components, along which the data vary the most. The first principal component captures the direction of maximum variance in the data.

Principal Components Regression (PCR) leverages this idea by constructing the first *M* principal components, and using them as predictors in a linear regression model fitted by ordinary least squares [63]. The underlying assumption is that a small number of principal components can often capture most of the variation in the original data and also reflect the relationship with the dependent variable *Y*. Although there is no guarantee that the directions of maximum variance in *X* are directly related to *Y*, it often turns out to be a reasonable enough approximation to fit X‐Y relationship [64].

We used the pcr function from the pls package to perform Principal Component Regression on the mtcars dataset. The code is as follows:

Algorithm 10 **Principal Components Regression (PCR).**

**Table:** 

library(pls) # For Principal Components Regression (PCR)
data(mtcars)
set.seed(123) # For reproducibility
# Split data into predictors (x) and response (y)
y <‐ mtcars$mpg
x <‐ mtcars[, ‐1] # Exclude mpg (response)
# Fit PCR with 10‐fold cross‐validation
pcr_model <‐ pcr(mpg ∼., data = mtcars,
scale = TRUE, # Standardize predictors
validation = "CV" # Cross‐validation
)
summary(pcr_model)
# Plot cross‐validated RMSE vs. components
validationplot(pcr_model, legendpos = "topright")
# Refit model with 3 components:
pcr_final <‐ pcr(mpg ∼., data = mtcars, scale = TRUE, ncomp = 3)
predicted <‐ predict(pcr_final, newdata = mtcars)
rmse <‐ sqrt(mean((predicted—mtcars$mpg)^2))
cat("RMSE:", rmse)
# Compare with Ordinary Least Squares (OLS)
lm_model <‐ lm(mpg ∼., data = mtcars) # Fit OLS model
# Compare coefficients
summary(lm_model) # Note potential multicollinearity issues
summary(pcr_final) # PCR coefficients are more stable

The results indicate that three principal components can explain 85.4% of the variance in the dependent variable, mpg. In comparison, an ordinary least squares (OLS) regression using all 10 explanatory variables explains 86.9% of the variance in mpg. However, due to severe multicollinearity, the regression coefficients from the OLS model are unstable.

#### 4.3.2 Partial Least Squares (PLS)

The PCR approach we just discussed involves identifying linear combinations, or directions, that best capture the variation in the predictor variables *X*. However, this is done in an unsupervised manner, meaning the response variable *Y* is not used in determining the principal component directions. In other words, the response does not guide the selection of these components. As a result, PCR has a key limitation: there is no guarantee that the directions which best summarize the predictors will also be the most useful for predicting the response [65].

Partial Least Squares (PLS) is a supervised alternative to PCR. Like PCR, PLS is a dimension reduction method that constructs a new set of features, the linear combinations of the original predictors, and then fits a linear regression model using these transformed features via least squares [66]. The crucial difference is that PLS identifies these new features in a supervised way, incorporating information from the response variable Y [66]. This allows PLS to find directions that not only capture the structure of the predictors but are also strongly related to the response [65]. In essence, PLS seeks directions that explain both the predictors and the response simultaneously.

We used the plsr function from the pls package to perform PLS on the mtcars dataset, which is the same data used for PCR. The code for PLS is as follows:

Algorithm 11 **Partial Least Squares (PLS).**

**Table:** 

# Fit PLS with 10‐fold cross‐validation
pls_model <‐ plsr(mpg ∼., data = mtcars,
scale = TRUE, # Standardize predictors
validation = "CV" # Cross‐validation
)

We used the same code as in PCR to refit the model using three components, and the resulting PLS model can explain 85.75% of the variance in the dependent variable mpg, which is slightly higher than the variance explained by PCR.

## 5 Conclusions

Whenever a researcher seeks to explain a response variable (such as growth rate, probability of survival, etc.) using multiple explanatory variables, a structured modeling approach can be highly effective. The first step is to apply random forest to assess the contribution of each explanatory variable and filter out irrelevant ones. Next, the variance inflation factor (VIF) should be used to detect multicollinearity, allowing for the removal of highly correlated variables. Subsequently, a full model incorporating all remaining variables, including two‐way interactions and quadratic terms, can be developed, followed by stepwise model selection to identify the most parsimonious model. The *MuMIn* package in R enables exhaustive comparison of all possible models, while *dominanceanalysis* package quantifies the relative importance of each variable across all multivariate contexts. Ridge regression and the lasso are advanced regularization techniques that improve model fitting by penalizing coefficient size to reach high model performance. Alternatively, dimension reduction methods, such as principal component regression (PCR) and partial least squares (PLS), do not eliminate irrelevant or correlated variables but transform them into a smaller set of uncorrelated components for use in regression. These methods are especially valuable when dealing with datasets containing many highly correlated predictors, such as the 19 bioclimatic variables representing temperature and precipitation [67].

## Author Contributions

B.L. analyzed the data, authored and reviewed drafts of the article, and approved the final draft. X.L. conceived the idea, analyzed the data, authored and reviewed drafts of the article, prepared figures and tables, and approved the final draft.

## Conflicts of Interest

The authors declare no conflicts of interest.

## Peer Review

The peer review history for this article is available in the Supporting Information for this article.

## Supporting information




**Supporting File 1**: ggn270027‐sup‐0001‐SuppMat_R_code.R.

**Supporting File 2**: ggn270027‐sup‐0002‐SuppMat_PeerReviewReport.docx.

## References

[1] P. McCullogh and J. A. Nelder , Generalized Linear Models, 2nd ed. (Chapman and Hall, 1989), 10.1007/978-1-4899-3242-6.

[2] A. C. Rencher and G. B. Schaalje , Linear Models in Statistics (John Wiley & Sons, 2008).

[3] G. James , D. Witten , T. Hastie , R. Tibshirani , and J. Taylor , “Linear Model Selection and Regularization,” An Introduction to Statistical Learning: With Applications in Python, eds. G. James , D. Witten , T. Hastie , R. Tibshirani , and J. Taylor (Springer International Publishing, 2023), 229–288.

[4] J. H. Zar , Biostatistical Analysis, 5th ed. (Pearson, 2010).

[5] Z. Zhang , E. Ersoz , C. Q. Lai , et al., “Mixed Linear Model Approach Adapted for Genome‐Wide Association Studies,” Nature Genetics 42 (2010): 355–360, 10.1038/ng.546. 20208535 PMC2931336

[6] J. P. Cook , A. Mahajan , and A. P. Morris , “Guidance for the Utility of Linear Models in Meta‐Analysis of Genetic Association Studies of Binary Phenotypes,” European Journal of Human Genetics 25 (2017): 240–245, 10.1038/ejhg.2016.150. 27848946 PMC5237383

[7] A. A. Shabalin , “Matrix eQTL: Ultra Fast eQTL Analysis via Large Matrix Operations,” Bioinformatics 28 (2012): 1353–1358, 10.1093/bioinformatics/bts163. 22492648 PMC3348564

[8] Y. Hai , J. Ma , K. Yang , and Y. Wen , “Bayesian Linear Mixed Model with Multiple Random Effects for Prediction Analysis on High‐dimensional Multi‐Omics Data,” Bioinformatics 39 (2023): btad647, 10.1093/bioinformatics/btad647. 37882747 PMC10627352

[9] M. E. Ritchie , B. Phipson , D. Wu , et al., “Limma Powers Differential Expression Analyses for RNA‐Sequencing and Microarray Studies,” Nucleic Acids Research 43(2015): e47. 25605792 10.1093/nar/gkv007 PMC4402510

[10] J. Straube , A.‐D. Gorse , P. C. E. Team , B. E. Huang , and K.‐A. L. Cao , “A Linear Mixed Model Spline Framework for Analysing Time Course ‘Omics’ Data,” PLoS ONE 10 (2015): e0134540, 10.1371/journal.pone.0134540. 26313144 PMC4551847

[11] M. D. Morris and T. J. Mitchell , “Exploratory Designs for Computational Experiments,” Journal of Statistical Planning and Inference 43 (1995): 381–402, 10.1016/0378-3758(94)00035-T.

[12] J. I. Daoud , “Multicollinearity and Regression Analysis,” Journal of Physics: Conference Series 949 (2017): 012009, 10.1088/1742-6596/949/1/012009.

[13] L. Breiman , “Random Forests,” Machine learning 45 (2001a): 5–32, 10.1023/A:1010933404324.

[14] S. J. Phillips and M. Dudik , “Modeling of Species Distributions with Maxent: New Extensions and a Comprehensive Evaluation,” Ecography 31 (2008): 161–175, 10.1111/j.0906-7590.2008.5203.x.

[15] X. Li , N. Li , B. Li , Y. Sun , and E. Gao , “AbundanceR: A Novel Method for Estimating Wildlife Abundance Based on Distance Sampling and Species Distribution Models,” Land 11 (2022): 660, 10.3390/land11050660.

[16] L. Breiman , “Statistical Modeling: the Two Cultures (With comments and a rejoinder by the author),” Statistical Science 16 (2001b): 199–215, 10.1214/ss/1009213726.

[17] S. Winham , X. Wang , M. de Andrade , et al., “Interaction Detection with Random Forests in High‐Dimensional Data,” Genetic Epidemiology 36 (2012): 142–142. 10.1186/1471-2105-13-164 PMC3463421 22793366

[18] M. A. Liaw , *Package ‘randomForest*’ (University of California, 2018).

[19] X. H. Li and Y. Wang , “Applying Various Algorithms for Species Distribution Modelling,” Integrative Zoology 8 (2013): 124–135, 10.1111/1749-4877.12000. 23731809

[20] F. Rohart , B. Gautier , A. Singh , and L. C. KA , “mixOmics: an R Package for 'Omics Feature Selection and Multiple Data Integration,” PLOS Computational Biology 13 (2017): e1005752, 10.1371/journal.pcbi.1005752. 29099853 PMC5687754

[21] J. Fox and S. Weisberg , An R Companion to Applied Regression (Sage Publications, 2018).

[22] J. J. Faraway , Extending the Linear Model With R: Generalized Linear, Mixed Effects and Nonparametric Regression Models, 2nd ed. (Chapman and Hall/CRC, 2016).

[23] C. Kooperberg , S. Bose , and C. J. Stone , “Polychotomous Regression,” Journal of the American Statistical Association 92 (1997): 117–127, 10.1080/01621459.1997.10473608.

[24] H. H. Zhang , G. Cheng , and Y. Liu , “Linear or Nonlinear? Automatic Structure Discovery for Partially Linear Models,” Journal of the American Statistical Association 106 (2011): 1099–1112, 10.1198/jasa.2011.tm10281. 22121305 PMC3222957

[25] X. Li , B. Li , G. Wang , X. Zhan , and M. Holyoak , “Deeply Digging the Interaction Effect in Multiple Linear Regressions Using a Fractional‐Power Interaction Term,” MethodsX 7 (2020): 101067, 10.1016/j.mex.2020.101067. 33072528 PMC7549115

[26] L. Engqvist , “The Mistreatment of Covariate Interaction Terms in Linear Model Analyses of Behavioural and Evolutionary Ecology Studies,” Animal Behaviour 70 (2005): 967–971, 10.1016/j.anbehav.2005.01.016.

[27] D. R. Campbell and N. M. Waser , “Genotype‐by‐Environment Interaction and the Fitness of Plant Hybrids in the Wild,” Evolution; International Journal of Organic Evolution 55 (2001): 669–676, 10.1554/0014-3820(2001)055[0669:GBEIAT]2.0.CO;2. 11392384

[28] J. F. Hair , W. C. Black , B. J. Babin , and R. E. Anderson , Multivariate Data Analysis (Pearson Education, Inc, 2009).

[29] R. R. Sokal and F. J. Rohlf , Biometry, 4th ed. (W. H. Freeman and Company, 2012).

[30] W. N. Venables and B. D. Ripley , Modern Applied Statistics With S (Springer Science & Business Media, 2013).

[31] J. A. Rice , Mathematical Statistics and Data Analysis (Thomson/Brooks/Cole Belmont, 2007).

[32] G. Casella and R. Berger , Statistical Inference (Chapman and Hall/CRC, 2024), 10.1201/9781003456285.

[33] S. W. Looney , Biostatistical Methods (Springer, 2002).

[34] A. F. Zuur , E. N. Ieno , N. J. Walker , A. A. Saveliev , and G. M. Smith , Mixed Effects Models and Extensions in Ecology With R (Springer, 2009).

[35] J. Royle and R. Dorazio , Hierarchical Modeling and Inference in Ecology. The Analysis of Data from Populations, Metapopulations and Communities (Elsevier Ltd., 2008).

[36] W. W. Stroup , M. Ptukhina , and J. Garai , Generalized Linear Mixed Models: Modern Concepts, Methods and Applications, 2nd ed. (Chapman and Hall/CRC, 2024), 10.1201/9780429092060.

[37] W. Härdle , Y. Mori , and P. Vieu , Statistical Methods for Biostatistics and Related Fields (Springer Science & Business Media, 2006).

[38] D. Siegmund and B. Yakir , The Statistics of Gene Mapping (Springer, 2007).

[39] S. Shen and J. A. Tuszynski , Theory and Mathematical Methods for Bioinformatics (Springer, 2008).

[40] F. M. Dekking , A Modern Introduction to Probability and Statistics: Understanding Why and How (Springer Science & Business Media, 2005).

[41] R. Knell . Biostatistics: Statistics Tutorials for Biologists (R package 2022), 10.32614/CRAN.package.Biostatistics.

[42] S. Wood , Generalized Additive Models: An Introduction With R (Chapman & Hall/CRC, 2006), 10.1201/9781420010404.

[43] A. Miller , Subset Selection in Regression (Chapman and Hall/CRC, 2002), 10.1201/9781420035933.

[44] C. Zuccaro , “Mallows' Cp Statistic and Model Selection in Multiple Linear Regression,” Market Research Society Journal 34 (1992): 1–10, 10.1177/147078539203400204.

[45] H. Bozdogan , “Model Selection and Akaike's Information Criterion (AIC): the General Theory and Its Analytical Extensions,” Psychometrika 52 (1987): 345–370, 10.1007/BF02294361.

[46] J. K. Nielsen , M. G. Christensen , and S. H. Jensen , “Bayesian Model Comparison and the BIC for Regression Models,” in 2*013 IEEE International Conference on Acoustics, Speech and Signal Processing* (IEEE, 2013), 6362–6366, 10.1109/ICASSP.2013.6638890.

[47] J. Shao , “Linear Model Selection by Cross‐Validation,” Journal of the American Statistical Association 88 (1993): 486–494, 10.1080/01621459.1993.10476299.

[48] L. A. Yates , Z. Aandahl , S. A. Richards , and B. W. Brook , “Cross Validation for Model Selection: a Review with Examples from Ecology,” Ecological Monographs 93 (2023): e1557, 10.1002/ecm.1557.

[49] D. Berrar , “Subset Selection in Encyclopedia of Bioinformatics and Computational Biology,” (Elsevier, 2019).

[50] K. P. Burnham and D. R. Anderson , Model Selection and Multimodel Inference: A Practical Information‐Theoretic Approach (Springer, 2002).

[51] R. Azen and D. V. Budescu , “Comparing Predictors in Multivariate Regression Models: an Extension of Dominance Analysis,” Journal of Educational and Behavioral Statistics 31 (2006): 157–180, 10.3102/10769986031002157.

[52] R. Azen and N. Traxel , “Using Dominance Analysis to Determine Predictor Importance in Logistic Regression,” Journal of Educational and Behavioral Statistics 34 (2009): 319–347, 10.3102/1076998609332754.

[53] W. Luo and R. Azen , “Determining Predictor Importance in Hierarchical Linear Models Using Dominance Analysis,” Journal of Educational and Behavioral Statistics 38 (2013): 3–31, 10.3102/1076998612458319.

[54] H. Wang and Y. Xia , “Shrinkage Estimation of the Varying Coefficient Model,” Journal of the American Statistical Association 104 (2009): 747–757, 10.1198/jasa.2009.0138. PMC2908045 20657760

[55] D. W. Marquardt and R. D. Snee , “Ridge Regression in Practice,” The American Statistician 29 (1975): 3–20, 10.1080/00031305.1975.10479105.

[56] A. M. E. Saleh , M. Arashi , and B. G. Kibria , Theory of Ridge Regression Estimation with Applications (John Wiley & Sons, 2019).

[57] T. Hastie and J. Qian , “Glmnet Vignette,” Retrieved June 9 (2014): 1–30.

[58] R. Tibshirani , “Regression Shrinkage and Selection via the Lasso,” Journal of the Royal Statistical Society Series B: Statistical Methodology 58 (1996): 267–288, 10.1111/j.2517-6161.1996.tb02080.x.

[59] P. Zhao and B. Yu , “On Model Selection Consistency of Lasso,” Journal of Machine learning research 7 (2006): 2541–2563.

[60] M. A. Carreira‐Perpinán , A Review of Dimension Reduction Techniques (Department of Computer Science. University of Sheffield, 1997), 1–69.

[61] Y. Ma and L. Zhu , “A Review on Dimension Reduction,” International Statistical Review 81 (2013): 134–150, 10.1111/j.1751-5823.2012.00182.x. 23794782 PMC3685755

[62] G. H. Dunteman , Principal Components Analysis (Sage, 1989), 10.4135/9781412985475.

[63] W. F. Massy , “Principal Components Regression in Exploratory Statistical Research,” Journal of the American Statistical Association 60 (1965): 234–256, 10.1080/01621459.1965.10480787.

[64] H. Artigue and G. Smith , “The Principal Problem with Principal Components Regression,” Cogent Mathematics & Statistics 6 (2019): 1622190, 10.1080/25742558.2019.1622190.

[65] P. D. Wentzell and L. V. Montoto , “Comparison of Principal Components Regression and Partial Least Squares Regression through Generic Simulations of Complex Mixtures,” Chemometrics and Intelligent Laboratory Systems 65 (2003): 257–279, 10.1016/S0169-7439(02)00138-7.

[66] J. Cha , “Partial Least Squares,” Advanced Methods in Marketing Research 407 (1994): 52–78.

[67] S. E. Fick and R. J. Hijmans , “WorldClim 2: New 1‐Km Spatial Resolution Climate Surfaces for Global Land Areas,” International Journal of Climatology 37 (2017): 4302–4315, 10.1002/joc.5086.

