# Supplementary material for: Best Practices for Developing Linear Models With Multiple Explanatory Variables
Source: Adv Genet (Hoboken). 2026 Feb 13;7(1):e00024. doi: 10.1002/ggn2.202500024 (PMC12902702; doi:10.1002/ggn2.202500024)
Supplement: Supplementary file 2 — Supporting File 2: ggn270027‐sup‐0002‐SuppMat_PeerReviewReport.docx. [file GGN2-7-e00024-s001.docx]

Record of Transparent Peer Review

ggn2.202500024

Best practices for developing linear models with multiple explanatory variables

Data submitted: 05-May-2025

Accepted: 18-Jan-2025

Please find below our point-to-point responses to the reviewers’ comments

**Responses to all comments**

- 26-Aug-2025: Pages 1-5
- 18-Dec-2025: pages 6-16
- 11-Jan-2026: page 17

**Aug.26, 2025**

Dear Dr Hu:

Thank you very much for organizing the review process and summarizing the reviewers' comments. The feedback provided is highly pertinent and constructive. We have made a substantial revision and responded to each comment below in blue font.

The manuscript provides a tutorial on variable selection in linear models with R, which will be helpful for biologists. However, reviewers raised concerns about the insufficient biological or genetics-related context, as well as the lack of clear objectives. To address these issues, we suggest including biology- or genetics-specific examples in the revision. Given its focus and structure, this manuscript is more appropriately categorized as a “Best Practices” review, and we recommend changing the article type into Review in the revised version.

Response: We replaced the original example datasets with those containing biological variables. In the first example, we employed a gene expression dataset instead of the previously car dataset and applied random forest to identify important variables. In the second example, we substituted the car dataset with animal longevity data to illustrate interaction effects. In the third example, we replaced the US crime data with tree diversity data to demonstrate the effect of a quadratic term. In the fourth example, we the gene expression dataset to replace the mtcars dataset, to show the stepwise model selection. In the newly added section, 4.1.7 Popular packages for model selection and variance partition, we used the gene expression dataset in the examples. For other examples (cross-validation, ridge regression, lasso, Principal Components Regression and Partial Least Squares), we continue to use the 'mtcars' and 'USArrests' datasets. There are three main reasons for this. First, both datasets are particularly well-suited for demonstrating these algorithms. Second, they are widely recognized and frequently featured in biostatistics and statistical learning textbooks. Third, both datasets are included in R's base datasets package, eliminating the need to install additional packages. Anyway, we can replace all dataset to biological associated datasets if it is necessary.

In the previous version, we did not explicitly highlight the objectives of this paper. In this revision, we have added a section, Section 1 Objectives, to clearly articulate the purpose and motivation for writing this paper.

Further, it is the author's responsibility to request permission to reproduce or adapt previously published figures. The copyright of figures generally belongs to the publisher of the journal where they first appeared. This also applies to your own previous publications. If you have not obtained permission to reproduce the figures yet, please do so before you submit your revised manuscript. We can only proceed with the publication once you have received all necessary permissions.

Response: All figures in this paper were newly created specifically for this publication. As such, we hold full rights to their use and publication.

Please provide a point-by-point response letter addressed to the reviewers, including a list of changes made and a rebuttal to any comments with which you disagree. You may copy the letter into the "Respond to Reviewers" box (if it is plain text only) or upload it as a "Response Letter to Reviewers" item (if it contains figures, tables, or special formatting such as formulas or equations). If necessary, you may also upload a separate revision cover letter addressed to the editor with any other information not intended for reviewers as a "Cover Letter to Editor" item. You will also be asked to upload a .zip archive containing the production data that will be used if your manuscript is accepted. See below for more details.

Response: We will submit three documents: a rebuttal letter with point-by-point responses to all comments, the manuscript with tracked changes, and the clean version of the manuscript.

------------------------------

REVIEWER REPORT:

------------------------------

COMMENTS TO AUTHOR:

Reviewer #1: This manuscript describes how to develop a linear model with multiple explanatory variables. The information described in this manuscript is useful to researchers attempting to perform statistical analyses. However, the following comments should be considered before accepting this manuscript.

Response: Thanks.

My comments are as follows:

1. Abbreviations1:

Although it is common in this field, it is not good to use only abbreviations such as ANOVA and ANCOVA.

Response: We have added the full names of ANOVA (Analysis of Variance) and ANCOVA (Analysis of Covariance) upon their first appearance in the manuscript.

2. Abbreviations2:

Some terms appear repeatedly with abbreviations such as AIC, BIC, RSS, n and k.

Response: We have added the full names and definitions of AIC, BIC, RSS, n, and k in the manuscript upon their first appearance.

3. Interaction or quadratic terms:

The authors describe that it is not recommended to include interaction terms or quadratic terms in dimensionality reduction regression. If so, the authors should indicate the reason.

Response: That is a very good question. Including interaction or quadratic terms before applying standard (linear) dimensionality reduction techniques like PCA is generally not recommended because it fundamentally alters the data structure by introducing non-linearity, which linear methods are not designed to handle effectively. This can distort the variance structure, lead to components that are difficult or impossible to interpret, and increase the risk of overfitting due to a sudden surge in feature dimensionality. Additionally, interaction and polynomial terms often have vastly different scales and variances compared to original variables, which can dominate the results of variance-based methods like PCA if not meticulously scaled. While non-linear dimensionality reduction methods (e.g., Kernel PCA, t-SNE) can capture such complex relationships more naturally, pre-adding interaction or quadratic terms is usually redundant and less effective than letting the algorithm discover non-linear patterns; therefore, it's typically better to perform dimensionality reduction on the original variables and incorporate non-linear effects in downstream modeling if needed.

4. Objectives and conclusions1:

This manuscript does not contain any objectives or conclusions in the main text. The reviewer cannot understand what the authors are trying to say with this manuscript.

Response: We have added a section on objectives as the first part of this manuscript to highlight the purpose of this paper.

5. Objectives and conclusions2:

If an article is intended to be published in this journal, the author should provide genetics-related cases to illustrate the point.

Response: We replaced the original example datasets with those containing biological variables. In the first example, we employed a gene expression dataset instead of the previously car dataset and applied random forest to identify important variables. In the second example, we substituted the car dataset with animal longevity data to illustrate interaction effects. In the third example, we replaced the US crime data with tree diversity data to demonstrate the effect of a quadratic term. In the fourth example, we the gene expression dataset to replace the mtcars dataset, to show the stepwise model selection. In the newly added section, 4.1.7 Popular packages for model selection and variance partition, we used the gene expression dataset in the examples. For other examples (cross-validation, ridge regression, the lasso, Principal Components Regression and Partial Least Squares), we continue to use the 'mtcars' and 'USArrests' datasets. There are three main reasons for this. First, both datasets are particularly well-suited for demonstrating these algorithms. Second, they are widely recognized and frequently featured in biostatistics and statistical learning textbooks. Third, both datasets are included in R's base datasets package, eliminating the need to install additional packages.

Reviewer #2: General comments

Authors present the appropriate practices of constructing linear models with multiple predictor variables. Among several potential predictor variables in hand, it could be a huge challenge for modelers to select the variables which do not have collinearity among themselves and significantly describe the variations of response variable. Authors briefly describe a number of approaches to select the appropriate predictor variables to construct linear models, such as machine learning approaches (e.g. random forest), stepwise selection approach (forward, backward, and hybrid), cross-validation, ridge regression, principal component analysis, lasso, etc. Authors also provide examples of the published data and results along with R-codes for each approach they have described. This manuscript seems to be somehow a structured summary of the statistical approaches of selecting variables for constructing linear models and evaluation of these models. Almost all the methods and approaches described in the manuscript are available in the standard statistical books and even in the research articles. Thus, this manuscript does not provide any novel knowledge to the potential audiences of the journal. All the approaches and methods of variables selection well-described can be found in the statistical books, and their applications have been used for a number of biological and physical researches. Instead of publishing this manuscript elsewhere, it could be beneficial for authors and audiences when they prepare a review article, exploring the existing statistical practices of variable selection and their pros and cons, especially in biological sciences, and get published. This, after making a good review article, it could be more innovative and interesting to audiences if authors would choose the appropriate journals that present the researches on the statistical methods and approaches, rather than submitting their works to the Advanced Genetics.

Response: We acknowledge that all the algorithms we have discussed have been in use for decades, and we did not introduce any novel methods. However, we observed that linear models are frequently misapplied, despite being well-established techniques. As the corresponding author of this manuscript, I have reviewed approximately 200 manuscripts and found that over 70% of them neglected interaction and quadratic terms in their linear models. This is a significant oversight. Having taught statistics for exactly 20 years, I have also noted that major textbooks worldwide often fail to emphasize the critical importance of these terms.

To address this gap, our primary innovation lies in proposing a “full model” framework that underscores the necessity of including interaction and quadratic terms, illustrated through practical examples. A secondary innovation is our recommendation to use random forest for preliminary variable selection, a method robust to multicollinearity, complex interactions, and higher-order effects.

This manuscript offers a systematic guide to model selection, providing best practices aimed at improving research methodology. Our goal is to correct the widespread misuse of linear models and promote more rigorous and accurate statistical modeling.

In the revised version, we added a section introducing the objectives of this paper, and highlighted our innovations in the section 3.	New insights for model selection (the sections of random forest and the full model framework were moved into this section).

Reviewer #4: This manuscript provides a nice review of linear models and a mini tutorial with the R code. The manuscript was well written and is concise. I believe this manuscript is useful for biologists. I only have a few minor editorial comments and a couple of suggestions as follows.

1. l.63, it should "regression."

Response: Thanks. We corrected this typo.

2. l. 112-113, although you cited a reference for avoiding the three-way interaction, it will be better that you use a couple of sentences to explain why we should not incorporate the 3-way interaction.

Response: That is a good suggestion. Two-way interaction terms should be considered because they capture meaningful and interpretable dependencies between pairs of variables, such as the effect of drugs depends on gender (drug a is good for men whereas drug b is good for women). In contrast, three-way interactions are often ignored because they are substantially more difficult to interpret, require larger sample sizes for reliable estimation, increase multicollinearity and overfitting risks, and rarely provide sufficient improvement in model fit to justify their added complexity. For example, a three-way interaction such as "the effect (Y) of drugs (X1) on men versus women (X2) depends on age (X3)" is often difficult to interpret and may lack clear practical significance. Unless there is strong theoretical justification, the marginal benefit of including three-way interaction is typically outweighed by the cost in clarity and stability, making two-way interactions the highest order of interaction routinely used in practice.

We added the example of the three-way interaction in the revision for clarity.

3. l. 313, To avoid confusion, I suggest using a subscript c for AIC_c. Consider the same for line 325.

Response: Thanks for the suggestion. We revised the AIC as AIC_least-square_. AIC_c is often used as a modified version of AIC that adjusts for small sample sizes and penalizes models with high dimensionality. We have done the same for BIC.

4. l. 342, a strength of your work is the R code for various models and accessible data sets from some R package, which provide some hands-on opportunities for authors to do along with their reading. Why not use the dredge() function in the R package MumIn to demonstrate how to use the metrics discussed in the section 5.1.5. You could use the same data from your example of 5.1.4.

Response: It is an excellent suggestion. We added one section, 4.1.7	Popular packages for model selection and variance partition, introducing the R packages *MumIn* and *dominanceanalysis* with examples and R code.

5. l. 612-613, I believe your work will be greatly improved if you add a concise summary paragraph here. For example, you could suggest your readers to design their linear models based on the biologically meaningful hypothesis so as to shorten the list of candidate variables. It is also good idea to compare PCR with other methods. For instance, it is difficult to interpret the results of PCR. However, PCR has advantages for prediction. I think it is also good to suggest the readers to screen multicollinearity using correlation matrix or variance inflation factor.

Response: Thanks for the suggestion. We added a conclusion section (210 words). We also added the process for checking multicollinearity using variance inflation factor, and provided R code in the section 3.1.

Xinhai Li, on behalf of all coauthors

**Dec. 18, 2025**

Dear Dr Hu:

Thank you very much for organizing the second-round review. The comments are highly pertinent and constructive. In particular, the final reviewer, an experienced statistician, not only provided exceptionally detailed and valuable suggestions, but also offered feedback on our responses to other reviewers’ comments. The comments help us substantially improved our manuscript.

We respond to each comment below in blue font.

COMMENTS TO AUTHOR:

Reviewer #1: The reviewer acknowledges that the authors have addressed most of the requested revisions. Nevertheless, the following points should be considered before the manuscript can be accepted.

Response: Thanks for your constructive comments.

My comments are as follows:

1. Fig. 1:

R code and plots appear abruptly in the text, but the manuscript does not explain which dataset was used or the purpose of the analysis.

Response: Thanks for this comment. We initially assumed that readers would be familiar with the R language and would follow the code in the order presented in the text. Figure 1, for instance, is referenced at line 105 of the R script. However, we recognize that the manuscript should be accessible to all readers, regardless of their prior experience with R. Accordingly, we have adopted the reviewer’s suggestion and added the following sentence to the main text: “The contributions of the variables (Fig. 1) and their partial effects (Fig. 2) can be visualized using the randomForest package.”

2. Figs. 2-4 & Tables 2-3:

Because Figs. 2-4 are not explained in the main text, it is difficult to understand what these figures and tables are intended to show.

Response: This is similar to the first comment. We have added sentences in the main text referring to Figures 2–4 and Tables 2–3.

Reviewer #2: Dear authors,

I went through the revised version of the manuscript, which has now been somehow improved, but not adequate enough to be published. I firmly stand with the issues raised in the first review. This manuscript needs to be converted to review article, and reasons for this are mentioned in the first review. Other reviewer has also suggested the same. Please consider converting this to review article through extensive literature analysis on statistical regressions, especially linear regression approaches and variable selections, which are applicable to genetics or quantitative analysis of biological phenomena.

Response: The primary aim of this manuscript is to present a best-practice framework for developing linear models. If the manuscript is transformed to a review, after citing maybe 100 literatures, we may reach results that some studies missed interaction terms, and some studies missed quadratic terms, etc. To the best of our knowledge, no existing work has implemented the integrated approach we propose. Many published studies perform exhaustive comparisons of numerous candidate models, yet none combine random forest–based variable selection with a full-model strategy for model building, let alone incorporate advanced techniques such as ridge regression, lasso, or dimension reduction methods.

Linear models have been a cornerstone of statistical analysis for over 90 years, yet a surprising number of errors persist in the literature, most notably the frequent omission of interaction and quadratic terms. We address this issue with theoretical justification, demonstrating the effects of interaction and quadratic terms and why these omissions can severely compromise model validity and inference. Transforming this work into a review would inevitably dilute these theoretical contributions, weakening the core message and methodological rigor that distinguish our approach.

Li and Li. Best practices for developing linear models with multiple explanatory variables MSS ggn2.202500024

Comments from quick read of unrevised draft.

Line 42 This approach provides an approximate estimation of the variance in Y, particularly useful when the exact mechanism underlying the relationship between Xs and Y is not fully understood. This statement is limited to additive (GLM) models. Generalized Linear Models (Table 1) handle multiplicative models, via log link.

Response: Generalized Linear Models use log link for Poisson regression and negative binomial regression, and logit link for logistic regression. All the transformation is applied to Y. We still use the linear combination of Xs to explain log(Y) or logit(Y). As such, our statement is applicable to all linear models including both general and generalized linear models.

Line 119 model selection techniques to eliminate insignificant terms. Use of statistical significance is not best practice for selection among multiple explanatory variables. Evidentialist (AIC) in place of frequentist avoids p-value criteria.

Response: We agree with this point. We revised the phrase as: “…eliminate terms that contribute little.”.

Line 542 In this section, we do not advocate for the inclusion of interaction or quadratic terms in the analysis.

State why not.

Response: Including interaction or quadratic terms before applying standard linear dimensionality reduction techniques, such as Principal Component Analysis (PCA), is generally discouraged. Doing so introduces non-linear relationships into the data, which fundamentally disrupts the assumptions underlying PCA. As a result, PCA may produce distorted representations of variance, yield components that are hard or impossible to interpret meaningfully, and become prone to overfitting due to the abrupt increase in feature dimensionality.

Moreover, interaction and polynomial features often exhibit vastly different scales and variances compared to the original variables. In variance-driven methods like PCA, these high-variance derived features can disproportionately dominate the principal components unless carefully standardized, a step that itself doesn’t fully resolve the conceptual mismatch between linear reduction and non-linear inputs.

While non-linear dimensionality reduction techniques (e.g., Kernel PCA, t-SNE, or UMAP) are better suited to capture complex structures, manually engineering interaction or quadratic terms beforehand is typically unnecessary and inefficient. These algorithms are designed to uncover such patterns inherently. Therefore, it is usually preferable to apply dimensionality reduction directly to the original, untransformed features and introduce non-linear effects later, during modeling, where they can be more flexibly and appropriately handled.

In this revised version, we added: “… because introducing non-linear relationships into the data fundamentally disrupts the assumptions underlying dimension reduction algorithms such as PCA.”.

Overall. No examples from genetics. Needs genetics examples or more appropriate journal. List of techniques, many of them long-known. Nothing new in list. Use of quadratic and interaction terms as alternative to eigenanalysis (PCA etc) appears to be novel.

Response: The revised manuscript has replaced most examples with genetic data. We have used section headings and subheadings to clarify the techniques we propose, and we believe this presentation is quite clear. The inclusion of quadratic and interaction terms is essential for all linear models, and it is not an alternative to eigenanalysis.

Objectives are not clear. “Best” but limited treatment of how to choose among the many techniques. Applicable to confirmatory as well as exploratory, if properly penalized criteria are used.

Response: It is true that the first version did not clarify the objectives. In the first revised version we added a dedicated "Objectives" section (the first section) to address this.

Revised MSS ggn2.202500024R1

In general the authors have addressed the reviewer comments appropriately and in detail. I note a few remaining problems relative to reviewer comments and author responses.

Reviewer #1 3. Interaction or quadratic terms: The authors describe that it is not recommended to include interaction terms or quadratic terms in dimensionality reduction regression. If so, the authors should indicate the reason.

The authors’ response appears to muddle two definitions of linear: additive effects (linear model) and straight-line (also called linear model). The first reason given misses the point. Eigenanalysis (dimension reduction) does not introduce non-linearity in the sense of additive effects. Rather, it imposes it with a new set of additive variables. By non-linear the authors appear here to mean additive effects, not straight-line regressions. Eigenanalysis captures covariance in the matrix of explanatory variables by inferring latent variables from observed variables. Canonical correlation is an example. . What eigenanalysis fails to do is disentangle covariance due to regression variables in a list of explanatory variables.

The distinction between eigenanalysis and techniques that partition variance needs to be made. That is, distinguish dimension reduction (cov(x1, x2...) from use of regression Y (Y | x1, x2…..)

Response: We highly appreciate the reviewer’s evaluation about our response to one comment from reviewer 1. Here, we must clarify the term “linear” and “linear models”. The term “linear” refers to both additive effects and straight-line effect. However, the “linear models” in statistics only means additive effect models, which use the linear combination of linear, quadratic, and interactional terms to represent additive effects of Xs on Y. Assuming linear models are straight-line models is a mistake, and it is the reason why many researchers missed the important quadratic terms and interaction terms.

Eigenanalysis such as PCA or canonical correlation uses linear combination of original variables to form a series ordered new variables called components. It naturally only quality linear relationship between original variables and derived components. Another ordination algorithm correspondence analysis can quantify unimodal relationship using chi-square distance based on reciprocal averaging. Eigenanalysis and linear models are not comparable because eigenanalysis such as PCA is a transformation of Xs (Y is not needed), and linear models quantify X-Y relationship.

Authors state:

vastly different scales and variances compared to original variables, which can dominate the results of variance-based methods like PCA if not meticulously scaled

This appears to refer to use of straight lines in PCA, instead of curves such as a quadratic. But maybe not. It is not clear.

Response: Here we explain the importance of standardization, which force the variance of all variables to 1.

There is no statement that this response to Reviewer #1 was added to the revision. This material is best left out of the revision.

Response: It is common practice that dimension reduction algorithms never include nonlinear terms such as quadratic and interaction terms. dimension reduction simply transforms variables, not explaining variances in Y.

Reviewer #2.

The comments of this reviewer matched my assessment: inappropriate data and well known techniques. The authors address the comment well.

Response: Thanks!

The authors state:

I have also noted that major textbooks worldwide often fail to emphasize the critical importance of these terms.

This appears to be missing from the revised manuscript. It is a worthwhile addition, keeping in mind that the topic of multiple explanatory variables is beyond the scope of major texts (i.e. used to teach stats).

Response: We used Hair’s influential textbook as a primary example and further reinforced our point by noting that two other widely respected texts, Biometry (4th ed., Sokal and Rohlf, 2012) and Biostatistical Analysis (5th ed., Zar, 2010), similarly do not emphasize the necessity of including interaction or quadratic terms.

I agree that the treatment of multiple explanatory variables remains largely beyond the scope of most mainstream statistics textbooks, particularly those used for teaching. This is indeed the current situation, and it is problematic: the majority of real-world analyses involve multiple explanatory variables, yet introductory and even intermediate textbooks have fallen significantly behind contemporary research practices.

Reviewer #4,

Authors’ response

three-way interactions are often ignored because they are substantially more difficult to interpret

This is not my experience. I do it every year for a course in stats for first year grad students in natural science. A non-negligible three-way term means that interactive effects of any pair depends on rhe third term. For factors, the analysis gets split by levels of the third term. For multiple regression the parameter estimate gets left in the model and can be interpreted as curvature.

Response: The meaning of a two-way interaction is crystal clear. For a three-way interaction, the most straightforward interpretation, as the reviewer noted, is that the strength or direction of the X₁×X₂ interaction changes depending on the level of X₃. In such cases, it becomes difficult (and often misleading) to make simple statements like “the relationship between X₁ and Y is positive or negative” or “Y is higher at level A of X₁ than at level B,” because those relationships depend on the values of the other interacting variables. Moreover, in linear models with additive effects, three-way interactions are generally unnecessary unless there is strong theoretical or empirical justification for their inclusion.

**Comments on revised manuscript.**

Line 12. The authors use the term exploratory here and at several points in the manuscript. The techniques here are only one of many techniques for EDA (Exploratory Data Analysis, Tukey 1977). Further, the techniques here are applicable in confirmatory settings.

Response: That is precisely what we mean. Exploratory research is characterized by flexibility, curiosity, and openness, and typically involves multiple variables, in contrast to experimental design, which often focuses on a limited set of controlled variables. Our manuscript is specifically oriented toward exploratory research.

The rationale for this review of techniques is still not clear in the revision (Objectives).

Response: The objectives were originally described in general terms, as we had omitted explicit mention of interaction and quadratic terms to avoid redundancy. However, we have now revised this section to explicitly include these terms.

Line 34 One key reason is that popular textbooks often fail to clarify the essential principles of linear model application.

It is not clear what this means, or how it relates to use of interaction and quadratic terms.

Response: A very common mistake in linear modeling is the omission of interaction and quadratic terms. This oversight often stems from inadequate training, as many standard textbooks themselves present an incomplete composition of model terms. In this paper, we aim to address and correct this widespread misconception perpetuated by conventional textbook approaches.

The topic of multiple explanatory variables is of interest in several fields, notably psychology and ecology. I have seen it appear in publications in biochemistry of lipids and metabolics. It certainly appears in multigene data. Identifying a genetics journal where multiple explanatory variables would be a worthwhile addition, showing relevance of the review to current practice in genetics research.

Response: Using multiple explanatory variables in linear models has long been the standard practice in exploratory studies across virtually all scientific fields. This approach is universally adopted in every major genetics journal, and we do not consider it necessary to identify a specific journal as evidence of this widespread convention.

The topic of multiple explanatory variables appears only briefly in standard texts. It is beyond the scope of introductory textbooks. I checked four advanced text books for examples of multiple explanatory variables.

Milliken and Johnson 2002. One example, with extensive treatment, confirmatory rather than exploratory.

Stroup 2007 no examples

Hardin and Hilbe 2012 no examples

Zuur Leno and et al 2007. Has many examples of multiple explanatory variables addressed by dimension reduction (eigenanalysis). Examples do not address quadratic and interaction terms.

Response: We greatly appreciate the reviewers’ efforts in examining four advanced textbooks for examples involving multiple explanatory variables. It is evident that mainstream textbooks rarely, if ever, clarify the importance of interaction and quadratic terms.

Recommendations

Add information from survey of 200 publications to introductory material.

Add survey of advanced texts, as above.

Response: This is not a review paper. Citing 200 studies and summarizing their use of linear models would add little value to the present work. Instead, we have included the 15 most widely cited textbooks and highlighted, in Table 2, how they address interaction and quadratic terms. Our analysis shows that these terms are either conflated with other nonlinear components or entirely omitted.

Line 35… another is that traditional approaches do not take advantage of modern machine learning algorithms.

Rationale well stated here.

Response: Thanks!

Line 36… 0 continous variables for logistic. Poisson, NB regression. How can rhis be? Perhaps this refers to regression routines applied to many categorical variables. Table 1 is inconsistent with respect to use of the word “regression.”

Response: In generalized linear models (GLMs), the concept of regression has been extended to settings where all predictors are categorical. For instance, a Poisson regression model that includes only categorical variables, such as log(E[Y])∼Gender * Country, is still correctly referred to as a Poisson regression. Such models are sometimes called log-linear models, particularly in the context of contingency table analysis, but the term Poisson regression is more widely used in modern applied statistics.

Line42. The correct citation is McCullagh and Nelder 2nd edition.

Response: We means general linear models here, so that our citation is:

Rencher, A. C., and G. B. Schaalje. 2008. Linear models in statistics. John Wiley & Sons.

However, we agree with the reviewer’s point that McCullagh and Nelder’s book on generalized linear models clearly addresses what they term the systematic component, the linear combination of explanatory variables. We have added the suggested citation here.

Line 62… multicollinearity becomes inevitable.

Only if there are covariates (continuous variables).

Response: Thanks! We added “continuous” in front of “explanatory variables”.

Line 123 typo

Response: Typo corrected.

Line 159… should not incorporate complex patterns such as three-way interactions, cubic terms, or higher-order terms

I cannot agree that three-way interactions fail to address relationship between explanatory variables and the dependent variable. This is certainly not true of models where the interaction term is the term of interest, such as presence (or not) of epistasis. Quadratic terms are one among many models for non-straight-line effects.

Response: We greatly appreciate the reviewer’s point and align himself with classical statisticians. In this paper, we aim to clarify that linear terms, two-way interactions, and quadratic terms are fundamental components of linear models, each carrying clear and interpretable meanings: a consistent positive or negative effect (linear), a conditional effect depending on another variable (two-way interaction), or a single maximum or minimum (quadratic). In contrast, cubic terms and three-way interactions lack straightforward interpretation; they can only be described in vague terms such as “inducing some change.” Linear models are not intended to capture highly detailed or complex X–Y relationships, and for most practical purposes, the inclusion of linear, two-way interaction, and quadratic terms is sufficient.

Line 166… the interaction effect reflects a non-additive relationship,

While this is true, it is routinely addressed by adding an interaction term Y ~ XA + XB + XA x XB This is a linear model because the 3 terms are taken as additive.

The material here confirms the comment above, on the confusion in the meaning of “linear model”

Response: Thanks for this comment. We changed “non-additive” to “nonlinear”.

Line 173 Quadratic terms in linear models represent a unimodal (U-shaped or anti-U-shaped) relationship between a predictor variable and the response variable

While quadratic terms are useful in representing local minimum or maximum relationships this is not always correct in practice. When a quadratic (i.e. parabolic) relation is fit, the two parameters can be such that a curve is fit with no minimum or maximum within the range of the data. The quadratic serves as a kluge to obtain residuals with no trend, but does not necessarily represent a relation with a minimum or maximum on a reasonable biological basis.

Response: We agree. The X-Y curve maybe only a section of a parabolic curve without a minimum or maximum point. We changed to “(U-shaped or anti-U-shaped, or a segment thereof)”.

This interpretation at line 173 recurs at line 270.

Response: The line numbers do not correspond to those in the previous version, and we did not identify the redundant content.

Lines 207-208. This phrasing is correct.

Response: Thanks!

The added material was not required by the reviewers. Reviewer #4 asked only for a “couple of sentences to explain why we should not incorporate the 3-way interaction.”

The added material is arguable and detracts from the manuscript.

Response: The added material uses an example to illustrate the complexity of three-way interactions. The central focus of this manuscript is to emphasize two-way interactions and deliberately exclude higher-order (e.g., three-way) interactions. We believe this addition is both important and necessary for clarity and interpretability.

Line 221… whether interaction terms in linear models are significant.

Use of statistical significance is not best practice for selecting model terms.

Avoid ‘significant’ unless clearly distinguished from hypothesis testing.

Authors need to warn against use of statistical significance where there no penalty for “researcher degrees of freedom.”

Response: We totally agree with this comment. We changed to “significantly contribute to the dependent variable”, focus on “contribute”.

Line 288 typo

Response: Typo corrected.

Line 341. In traditional best subset selection, the standard approach involves comparing models that include every possible combination of explanatory variables, along with their two-way interaction terms.

Response: Yes, traditional best subset selection involves comparing models that include every possible combination of terms (linear, quadratic, cubic, and high order interaction).

The criterion used matters. P-values, as in the past, are poor practice.

Response: We agree that p-values are not well suited for model selection, as they are highly dependent on sample size. Nevertheless, p-values remain the most widely used metric for hypothesis testing and model development. The debate surrounding their use has persisted for over 60 years and continues to this day.

The authors need to explain the importance of the default criterion, AIC, for step().

It needs to be mentioned here, even though this is covered to some degree in subsequent material. The authors need to explain why the result of these model selection procedures, while useful, do not produce a model with any predictive capacity.

Response: In Section 4.1.4, we introduced the step() function, mentioned the default criterion, AIC. In Section 4.1.5	Model performance indices: Cp, AIC, BIC, and Adjusted R^2^, we introduced AIC in a theoretical manner. In the previous Section 4.1.1 Comparison with all potential models, we introduced the whole model selection option. We think it is already clear that step() using AIC does not produce a model with any predictive capacity.

Line 406. Explain “training data.” when the term first appears.

Response: Thanks for this comment. We added “(the observed data)” after “training data” for clarification.

Table 2. This table has many coefficients and model fit indices.

The reader is left with no idea how to use delta, or what it is.

Note that the use of AIC here is contrary to what Burnham and Anderson advocate for AIC. This distinction needs to be cleat. State how the unstructured use here differs from Burnham and Anderson and why the use here is justifiable in an exploratory setting, and in a confirmatory setting The manuscript uses the term model selection. The manuscript would benefit from a short statement of how information theoretic (AIC) model selection (Burnham and Anderson) differs from model selection as described in this manuscript. Note, as above, that the techniques listed in this manuscript are applicable to confirmatory analyses

Response: Actually, we focus on exploratory analyses which have numerous explanatory variables to be selected. We introduce the techniques for exploratory analyses, not for confirmatory analyses. Burnham and Anderson do not argue that AIC is unsuitable for exploratory analyses, quite the opposite, they strongly advocate for the use of AIC precisely in the context of exploratory data analysis and multimodel inference, especially when there is no single, well-established theory dictating a unique model. In their influential book Model Selection and Multimodel Inference: A Practical Information-Theoretic Approach (2nd ed., 2002), Burnham and Anderson promoted AIC as a tool for scientific discovery, particularly in exploratory settings where multiple competing hypotheses (represented as statistical models) exist. They emphasized model comparison and weighting (via AIC weights) rather than selecting a single "best" model, this is inherently an exploratory and inferential approach.

Table 3 has many values of R^2

How does the reader interpret the result?

Which matrix is used? the upper triangular? lower? both?

Response: The table should be read vertically to assess the contribution of each individual term, or horizontally to examine the contribution of terms under a specific condition (i.e., within the model shown in that column). Unlike a correlation matrix, it does not have upper or lower triangular symmetry and should not be interpreted as such.

Line 596 Choosing an appropriate value of λ is crucial for achieving optimal performance, How does the reader do this.?

Response: That is a good question. We added: “The cv.glmnet() function in the glmnet package selects the optimal λ through k-fold cross-validation: it fits the model across a grid of λ values, computes the average prediction error (typically mean squared error) for each λ by training on k−1 folds and validating on the held-out fold, then chooses either lambda.min, the λ with the lowest cross-validated error, or lambda.1se, the largest λ within one standard error of the minimum, favoring a more regularized (less overfit) model.”.

Lines 321 – 571. Clear explanation of successive improvements on long known techniques.

Response: Thanks!

Line 611. Use “additive” not “linear” to reduce confusion due to two meanings of “linear.”

Response: Again, due to the line number problem, we can not locate the “linear”. Please see above response, “linear model” has a unique definition, including nonlinear terms.

Line 710. Dimension reduction has long been common as the method of choice (e.g. Zuur et al). It is very different from backwards/forwards selection and the many techniques listed in this manuscript. Eigenanalysis replaces the set of explanatory variables with uncorrelated latent variables. The distinction can be made clear by treating eigenanalysis first.

Response: Yes, both shrinkage methods and dimension reduction are advanced model selection techniques. Our goal is to present a best-practice workflow that progresses from basic to advanced approaches. Rather than delving into the theoretical underpinnings of eigenanalysis, we focus on providing practical methods and ready-to-use R code that readers can apply directly.

Line 720. Eigenanalysis combines variables to obtain new variables. The original variables are replaced, not transformed. This is one of the limitations of eigenanalysis.

Response: We agree with this point. Another way to understand this is that the derived components are linear combinations of the original variables, with the eigenvectors serving as the transformation coefficients.

Line 775 Singular noun takes singular verb.

Response: Grammar error corrected.

Lines 710 -792. Generally clear and well presents, except as above. Because eigenanalysis is widely used, this material is better placed before the more advanced material. A short summary sentence on limits of eigenanalysis would be appropriate: e.g. it includes all variables This change would entail revision of lines 805-809.

Response: Thank you for your comments. We agree that eigenanalysis is widely used, and it is simple and transparent. However, using eigenanalysis to perform further Principal Components Regression (PCR) and Partial Least Squares (PLS) is more advanced. Anyway, eigenanalysis itself is a distinct algorithm and, while valuable, is not central to our discussion; therefore, a detailed introduction is not warranted here.

Summary.

The objectives section is incomplete with respect to rationale.

The two meanings of “linear” need to be disentangled.

The techniques here are only one of the many forms of Exploratory Data Analysis (EDA Tukey 1977). The techniques here are not limited to exploratory analyses, they are used in confirmatory (hypothesis testing) applications. The AIC techniques here are contrary to AIC model selection in Burnham and Anderson.

The authors are encouraged to address these items, or find an applied statistician who can address these items and the applicability of these techniques to model selection in exploratory and confirmatory analysis. The iterative practices in data science are inconsistent with best practice in natural and social science settings.

"Let the computer find out" is a poor strategy for researchers who do not bother to think clearly about the problem of interest and its scientific setting.

p117 in Burnham, K.P., D.R. Anderson. 1998. Model Selection and Inference.

Response: It is so nice that the key comments were reiterated here. We have addressed all of them thoroughly and thoughtfully. It is evident that the reviewer is an experienced statistics educator. I have taught statistics for 20 years, and I deeply value the reviewer’s insightful and detailed feedback. Most importantly, the reviewer embodies the perspective of traditional statisticians whom we respectfully engage with, and at times gently challenge, through the novel ideas presented in this paper. The dialogue with the reviewer thus reflects the very core of our work’s intellectual contribution.

Xinhai Li, on behalf of all coauthors

**Jan. 11, 2026**

Dear Dr. Hu:

Thank you very much for informing me that the manuscript has been recommended for publication pending further revisions. We greatly appreciate your thoughtful suggestion and for providing 10 references on linear models for our consideration. I have carefully read all 10 papers and have cited six of them in the revised manuscript. These works present advanced methods with more complex algorithms, whereas our study focuses on fundamental linear models. Nonetheless, including these references significantly enriches the context and scholarly foundation of our paper.

Xinhai
